# Online Coupling of 80 MHz Benchtop 2D‐COSY NMR to HPLC

**DOI:** 10.1002/marc.202500239

**Published:** 2025-05-18

**Authors:** Marianne Gaborieau, Markus Matz, Johanna Tratz, Michael Pollard, Manfred Wilhelm

**Affiliations:** ^1^ Institute of Chemical Technology and Polymer Chemistry Karlsruhe Institute of Technology (KIT) Engesserstraße 18 76131 Karlsruhe Germany

**Keywords:** 2D‐NMR, compact NMR, correlation spectroscopy (COSY), HPLC‐NMR, non‐uniform sampling (NUS)

## Abstract

HPLC is coupled online for the first time to 2D‐NMR on a benchtop NMR spectrometer. The potential is demonstrated at 80 MHz with COSY (^1^H‐^1^H COrrelation SpectroscopY) for a mixture of methyl and propyl parabens, additives commonly used in cosmetics. To lessen the impact of short residence time in the NMR flow cell, a repetition delay reduction combined with non‐uniform sampling (NUS) allows decreasing the COSY experiment duration from 1 h to 2 min. The HPLC‐benchtop 2D‐NMR online coupling is established for the first time, using three different modes: stop‐flow (with short stops at the analyte peaks and in the baseline), slow‐flow (with a lower, constant flow rate over the whole elution range of analytes) and steady‐flow (with a constant flow rate throughout the experiment). The HPLC‐benchtop 2D‐NMR online coupling allows the successful separation and characterization of methyl and propyl parabens at low field. This provides more in‐depth chemical information on separated analytes than HPLC‐benchtop 1D‐NMR, with a smaller footprint, reduced experimental complexity and at lower costs than HPLC‐high‐field 2D‐NMR. It opens the way to other 2D‐NMR experiments and the characterization of a wider range of analytes such as oligomers, small molar mass additives, or polymers.

## Graphical abstract

The online coupling of 2D‐NMR to HPLC is demonstrated for the first time with benchtop NMR. COSY spectra of parabens are recorded at 80 MHz, with stop‐flow (with short stops at analyte peaks and in baseline), at slow‐flow (with lower, constant flow rate over the analytes elution range) and steady‐flow (at constant flow rate throughout the experiment).

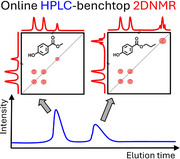

## 1 Introduction

Combining different analytical techniques is required to address the growing complexity of sample mixtures including small and large analytes, additives, and contaminants. The hyphenation of spectroscopy to chromatography therefore combines a separation technique to a detection providing chemical information.^[^ ^1^, ^2^, ^3^ ^]^ It yields chemical information on the analytes eluting at each point of the chromatogram and provides a physical separation of the analytes from mixtures before their spectroscopic characterization. The detection includes mass spectrometry,^[^ ^4^, ^5^ ^]^ infrared^[^ ^6^, ^7^, ^8^ ^]^ or NMR spectroscopy.^[^ ^9^, ^10^, ^11^, ^12^ ^]^ Among them NMR spectroscopy yields unique insights into molecular structures and allows quantitative investigations.

The online coupling of chromatography with NMR spectroscopy was initially developed with large NMR instruments. It was first reported more than four decades ago with bulky electromagnets: originally in stop‐flow mode by Watanabe and Niki in 1978^[^ ^13^ ^]^ and for the first time on flow by Bayer et al. in 1979.^[^ ^14^ ^]^ The technique took off later with superconducting, high‐field NMR spectrometers^[^ ^15^ ^]^ for applications as diverse as biomedical, pharmaceutical and environmental studies or natural products^[^ ^1^ ^]^ as well as oligomers and polymers.^[^ ^10^, ^16^, ^17^ ^]^ It is now widely established.

Compact, benchtop NMR spectrometers based on permanent magnet arrays, are more affordable than their high‐field counterparts based on superconducting magnets. They have been commercial for about 15 years and currently reach a magnetic field strength of about 2 Tesla.^[^ ^18^ ^]^ This coincided with the research into HPLC‐NMR with compact NMR instruments, starting with prototypes^[^ ^19^ ^]^ and now taking advantage of commercial instruments,^[^ ^20^ ^]^ for polymer samples with size‐exclusion chromatography (SEC). Benchtop NMR spectrometers operate at lower magnetic fields and are therefore less sensitive than high‐field instruments. One advantage is the horizontal static magnetic field that allows for sensitive coils. Steady‐flow SEC‐benchtop NMR thus necessitated an in‐depth optimization (of HPLC, NMR, and data processing parameters) on model polymers to increase its sensitivity and allow access to spectrally‐resolved chromatograms with sufficient signal‐to‐noise ratio (*SNR*) during the analytes’ elution.^[^ ^11^ ^]^ This enabled its application to real‐life samples such as synthetic rubbers,^[^ ^21^ ^]^ industrial polymer blends or gradient copolymers and surfactant blends.^[^ ^22^ ^]^ It has recently been extended to liquid‐adsorption chromatography (LAC) through LAC‐benchtop NMR of alkyl parabens, preservatives commonly used in cosmetics, enabling their separation and quantification below the legal limit of 4 g L^−1^ in mixtures.^[^ ^12^ ^]^

2D‐NMR spectroscopy is more powerful than 1D‐NMR spectroscopy for the structural elucidation of unknown compounds or compounds with small structural variations, as it yields information on the spatial relationships between nuclei.^[^ ^23^, ^24^, ^25^ ^]^ 2D‐NMR spectra typically take much longer to record than 1D‐NMR spectra as they are normally recorded as series of consecutive 1D experiments with variable evolution times incremented between individual 1D experiments (referred to as increments in the indirect dimension). 2D‐NMR was thus initially hyphenated to HPLC at high field and with peak parking or stopped flow to afford sufficient time to record individual 2D ^1^H NMR spectra of the different analytes (in more than 10 h).^[^ ^26^, ^27^ ^]^ Alternative 2D‐NMR experiments were then designed to speed up the recording of 2D‐NMR spectra. Ultrafast 2D‐NMR spectroscopy employs spatial encoding to record simultaneously all the increments for the indirect dimension in one scan.^[^ ^28^ ^]^ It was used for chromatography‐NMR(2D) under on‐flow conditions, reported first using a home‐made apparatus with a pressurized silica‐based glass column, recording TOCSY 2D‐NMR spectra in 37 s in a non‐protonated solvent CCl_4_,^[^ ^29^ ^]^ then with commercial HPLC‐high‐field NMR instrumentation, recording COSY 2D‐NMR spectra in 12 s using a partially deuterated solvent.^[^ ^30^ ^]^ It relies on the analyte signal being sufficiently strong, as the sample in the detection volume is virtually fractionated to record different indirect dimensions increments.^[^ ^31^ ^]^ Hadamard 2D‐NMR relies on multiplexing and irradiation at multiple radiofrequencies to construct the full 2D‐NMR spectrum based on a limited number of scans.^[^ ^32^ ^]^ It was used for HPLC‐NMR(2D) on‐flow with commercial HPLC‐high‐field NMR instrumentation, recording TOCSY 2D‐NMR spectra in 15–25 s in a non‐deuterated solvent.^[^ ^33^ ^]^ It requires a priori knowledge of the resonances of the analytes.^[^ ^31^ ^]^ To our knowledge the online coupling of 2D‐NMR to HPLC was only reported with high‐field NMR spectrometers.

HPLC‐NMR(2D) is extended here to benchtop NMR as an affordable solution to yield in‐depth chemical information on analytes. The 2D‐NMR experiment COSY (^1^H‐^1^H COrrelation SpectroscopY), historically the first 2D‐NMR experiment proposed^[^ ^23^, ^24^ ^]^ was selected for its ability to be carried out under flow on a benchtop NMR spectrometer^[^ ^34^ ^]^ and its capability to reveal connectivity in analyte molecules. The possibilities of the online coupling of benchtop 2D‐NMR to HPLC are explored here with stop‐flow, slow‐flow, and steady‐flow using alkyl parabens^[^ ^12^ ^]^ as model compounds in non‐deuterated solvents.

## 2 Results and Discussion

### 2.1 COSY of Parabens and Considerations for Online Coupling

**Figure 1:**
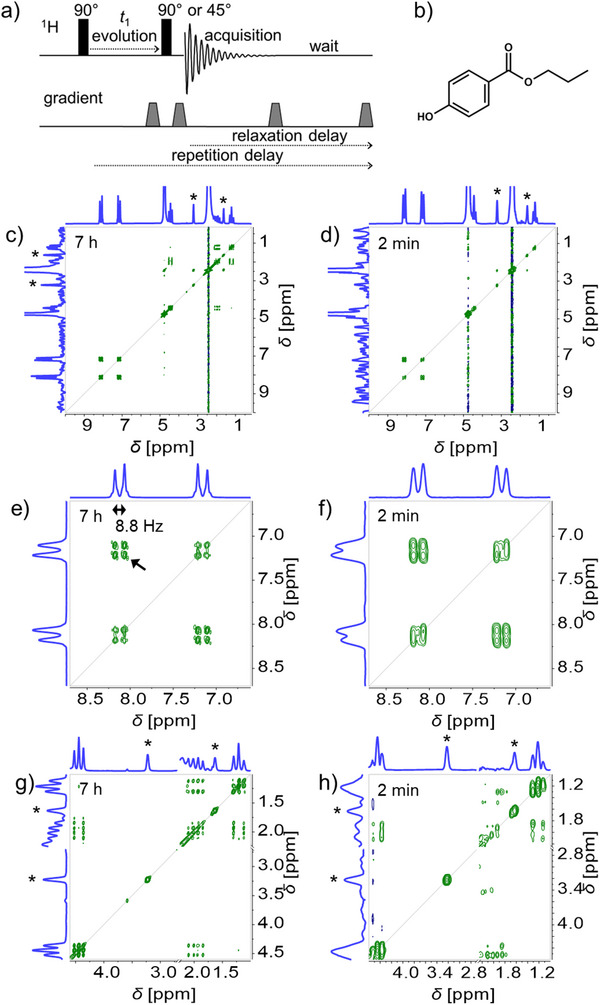
Pulse scheme (a), molecular structure (b) and COSY spectra (c–h) for propyl paraben (30 g L^−1^ in acetone/water 60/40 v/v) at 0.0 mL min^−1^ with circulatory setup (stock solution flowing through HPLC pump and NMR flow cell, without HPLC column, in this case the flow was stopped for the measurement). COSY spectra are shown as a reference COSY spectrum before enhancement (c, e, g, with 5∙*T*_1_^*^ relaxation delay, 4 scans, 512 increments in the indirect dimension, in 7 h 15 min), and after enhancement (d, f, h, at Ernst condition, with 1 scan, 256 increments in the indirect dimension, 37.5% NUS density, in 1 min 54 s). COSY spectra are shown at full 0–10 ppm scale (c, d), covering the aromatic region (e, f), and the aliphatic region (g, h, with acetone signal cut out for display through an axis break at 2.25–2.70 ppm). The arrow in e) indicates the cross‐peak used for *SNR* assessment (Section 2.2). * indicates an acetone signal ^13^C satellite.

The pulse scheme for the gradient‐selected COSY experiment chosen in this work is shown in **Figure** **1**a. It was selected over faster schemes like ultrafast COSY or Hadamard COSY for its sensitivity and its simplicity. Since oxygen content and flow affect the apparent relaxation of functional groups (longitudinal relaxation *T*_1_ and transverse relaxation *T*_2_)^[^ ^35^ ^]^ the refinement of COSY experimental conditions was carried out with a circulatory setup on a stock solution that flowed through the HPLC pump and degasser at various flow rates. The analyte chosen for this improvement was propyl paraben at 30 g L^−1^ (see Figure 1b for molecular structure). All experiments were conducted in a non‐deuterated solvent (acetone/water 60/40 v/v). Flow rates were selected as follows: 1.0 mL min^−1^ as the flow rate of previous steady‐flow HPLC‐NMR experiments,^[^ ^12^ ^]^ 0.4 mL min^−1^ as a lower flow rate to broaden HPLC peaks on the elution time scale and afford a longer time to record COSY spectra in HPLC peaks, as well as 0.0 mL min^−1^ to reflect stopping the mobile phase flow in HPLC peaks (or baseline) to adjust the time available to record COSY spectra. The apparent *T*_1_^*^ values recorded for the aromatic ^1^H NMR signals of propyl paraben in these conditions at different flow rates (as well as in a standard 5 mm NMR tube without degassing) are shown in Figure S3 (Supporting Information). They range from 1.1 to 2.5 s.

A reference COSY spectrum of propyl paraben (30 g L^−1^ in acetone/water 60/40 v/v) is shown in Figure 1c,e,g (see also Figure S4, Supporting Information). It was recorded with 512 increments in the indirect dimension and 4 scans to ensure high sensitivity and well‐defined line shapes. It exhibits two doublets at 8.1 and 7.2 ppm with an 8.8 Hz J‐coupling in the aromatic region, a methyl group (triplet) at 1.2 ppm, and methylene groups (triplet and quartet of triplets) at 4.4 and 2.0 ppm (overlapping with the base of solvent signals). The main drawback of 2D‐NMR spectroscopy is the long time typically needed to record spectra, with 7 h needed to record this intense COSY spectrum. This is much too long to consider for online coupling with HPLC. A 2‐min target was set in this work as a practical goal for the maximal COSY experiment duration. The number of increments in the indirect dimension was first reduced to 256 (minimal number to preserve the 8.8 Hz splitting pattern of aromatic nuclei) and the number of scans to 1, bringing the COSY experiment duration down to 54 min (see Figure S5a,b, Supporting Information). An additional enhancement of NMR parameters (repetition time, pulse angle and non‐uniform sampling) was then carried out to further decrease the COSY experiment duration.

### 2.2 COSY Experiment Enhancement

#### 2.2.1 Enhancing Sensitivity and Experiment Duration Through the Repetition Delay

The sensitivity in NMR spectroscopy scales with the square root of the number of scans when signal averaging is applied,^[^ ^36^ ^]^ hence the sensitivity of the COSY experiment with different settings is assessed here as *SNR* divided by the square root of the total experiment duration (referred to as intrinsic sensitivity). The signal chosen for this assessment is the cross‐peak at 8.05,7.20 ppm in the COSY spectrum of propyl paraben (see arrow in Figure 1e). The precision of the sensitivity measurement is assessed through the standard deviation of five repeat measurements of the same sample. The reference situation chosen here is the “5∙*T*_1_^*^” condition in which a 90° pulse irradiation is used and the repetition time is five times the apparent *T*_1_ relaxation time, leading to quantitative spectra under stop‐flow conditions.^[^ ^36^ ^]^ To speed up acquisition, a shorter repetition delay was also employed; the highest sensitivity under steady state with a 90° pulse irradiation is then observed for a relaxation time of 1.3∙*T*_1_^*^ (referred to as “1.3∙*T*_1_^*^” condition here). It is also possible to reduce both the nutation angle *α* and the repetition delay, with the optimal sensitivity in the steady state then observed for a repetition delay of ‐ln(cos(*α*))∙*T*_1_^*^; the optimal nutation angle for a given repetition delay is called the Ernst angle. Here, a 2nd irradiation pulse flip angle was chosen at 45° with a corresponding relaxation time of ‐ln(cos(45°))∙*T*_1_^*^ (≈0.35∙*T*_1_^*^, referred to as “Ernst” condition). This is the COSY‐45 experiment, designed for reducing the diagonal signals and increasing the resolution with faster pulsing.^[^ ^37^ ^]^

**Figure 2:**
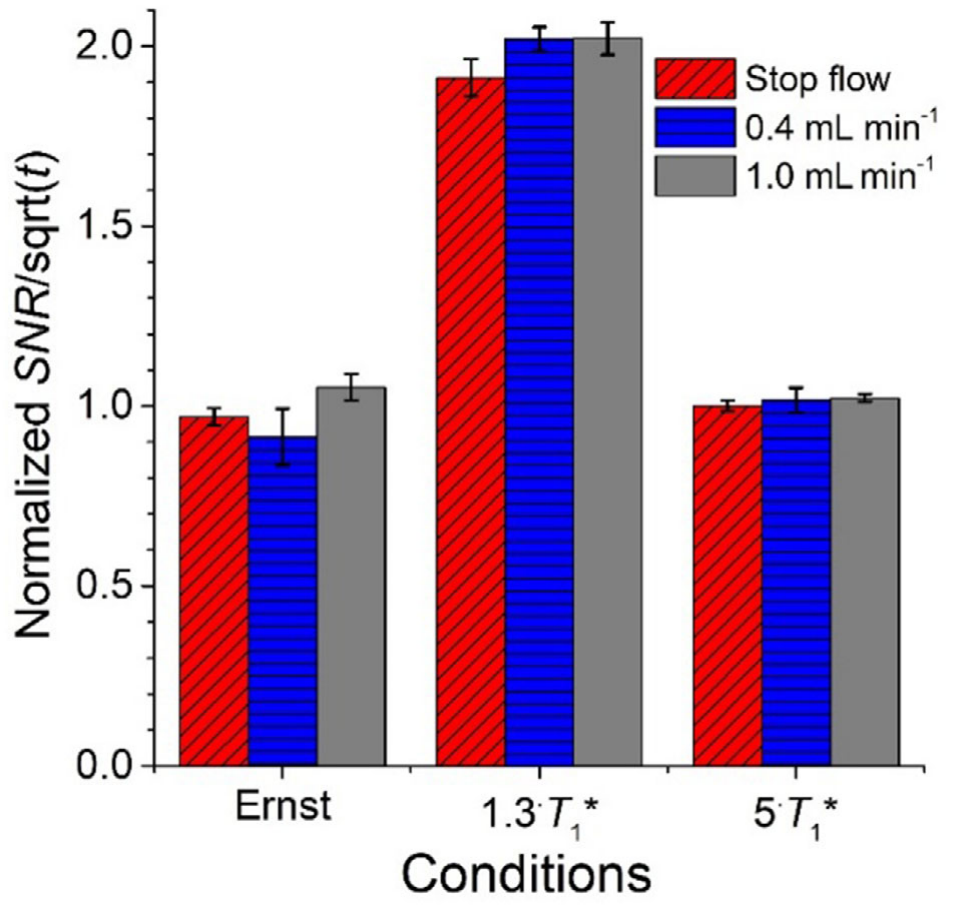
Signal‐to‐noise ratio per square root of experiment duration for the cross‐peak at 8.05,7.20 ppm in the COSY spectrum of propyl paraben (30 g L^−1^ in acetone/water 60/40 v/v) measured at different flow rates with a circulatory setup and normalized to the value determined for 5∙*T*_1_^*^ relaxation delay at 0.0 mL min^−1^. See Table S1 (Supporting Information) for relaxation delays, experiment durations and *SNR* values at 0.0 mL min^−1^.

At each flow rate (0.0, 0.4, and 1.0 mL min^−1^) the same trend was observed: the highest intrinsic sensitivity was observed at the 1.3∙*T*_1_^*^ condition, and similar intrinsic sensitivities were observed at the 5∙*T*_1_^*^ and Ernst conditions (see Table S1, Supporting Information for *SNR* values at 0.0 mL min^−1^ and **Figure** **2** for all values of *SNR* divided with the square root of experiment duration then normalized to the value determined for 5∙*T*_1_^*^ relaxation delay at 0.0 mL min^−1^). The shortest experiment duration was obtained for the Ernst condition, without compromising the intrinsic sensitivity (Figure 2) or the resolution (Figure S5c,d compared to S5a,b, Supporting Information) compared to quantitative conditions. The COSY experiment duration at the Ernst condition was 3.5–5 min depending on the flow rate, a duration much shorter than the initial 54 min but still significantly larger than the 2 min practical target.

#### 2.2.2 NUS to Reduce Experiment Duration

Non‐uniform sampling (NUS) consists in decreasing the experiment duration by recording only some of the increments in the indirect dimension while retaining the same total acquisition length in the indirect dimension to preserve the resolution (Figure S6, Supporting Information).^[^ ^38^, ^39^ ^]^ The full signal is then reconstructed to compensate for the missing data. It is characterized by the NUS density, the percentage of increments actually recorded.

COSY spectra of propyl paraben were recorded at 0.4 mL min^−1^ at the Ernst condition with NUS densities of 10%, 25%, 37.5%, 50%, 75%, and 100% of 256 increments in the indirect dimension (Figures S7 and S8, Supporting Information); these correspond to experiment durations of 40 s to 5 min (Figure S11, Supporting Information). For high NUS densities (37.5% and above here) the COSY spectra are similar to the ones without NUS, providing a reduction in experiment duration without compromising the quality of the spectra recorded. For low NUS densities (10 and 25% here) the number of data points recorded is insufficient to reconstruct the full 2D pattern of the COSY spectrum, leading to information loss, seen for example in baseline distortions and on the fading/disappearance of cross‐peaks in the aliphatic region. It was decided to proceed with a NUS density of 37.5% of 256 increments (corresponding to 96 data points in the indirect dimension) as this is the minimal density for which no distortions are observed in the spectra, in particular the cross‐peaks of the alkyl side chain are still observed (Figure S8, Supporting Information).

The number of increments in the indirect dimension was then reduced from 256 to 128 (corresponding to 48 data points in the indirect dimension) in an attempt to reduce the experiment duration by a further factor of 2. At 128 increments, a similar behavior was observed with a reduction in NUS densities as for 256 increments (see Figures S9 and S10, Supporting Information). However, the resolution in the indirect dimension was insufficient to resolve the splitting pattern of the aromatic signals, with at best only a faint shoulder observed. It was thus decided to proceed with 256 increments in the indirect dimension.

The enhanced conditions determined here were thus COSY‐45 with Ernst condition for the repetition delay, a NUS density of 37.5% of 256 increments in the indirect dimension and 1 scan, resulting in an experiment duration of 2 min. These fulfill the 2 min experiment duration target set for online coupling with HPLC and were used for all coupling experiments (Sections 2.3 – 2.5). Three options were explored for this online coupling, keeping in mind the 2 min needed to record individual COSY spectra on eluting analytes: i) stopping the flow for a few min to record COSY spectra at specific elution volumes (Section 2.3), ii) slowing down the flow over the elution range of analytes to broaden the chromatographic peaks on the elution time scale (Section 2.4) and iii) simply at steady‐flow carrying out the whole chromatographic separation at a constant flow rate (Section 2.5). It takes advantage of background work on flow programming in HPLC‐NMR(1D) carried out in our team to set up and understand stop‐flow and slow‐flow (in preparation for publication). The HPLC separation was carried out following earlier work^[^ ^12^ ^]^ with a C18 semi‐preparative column, injecting 1 mL of paraben solutions at 50 g L^−1^ in acetone/water 60/40 v/v, using acetone/water 60/40 v/v as the mobile phase.

### 2.3 Online Coupling of HPLC and 2D‐NMR with Stop‐Flow

#### 2.3.1 Stop‐Flow 2D‐NMR Detection of Propyl Paraben

**Figure 3:**
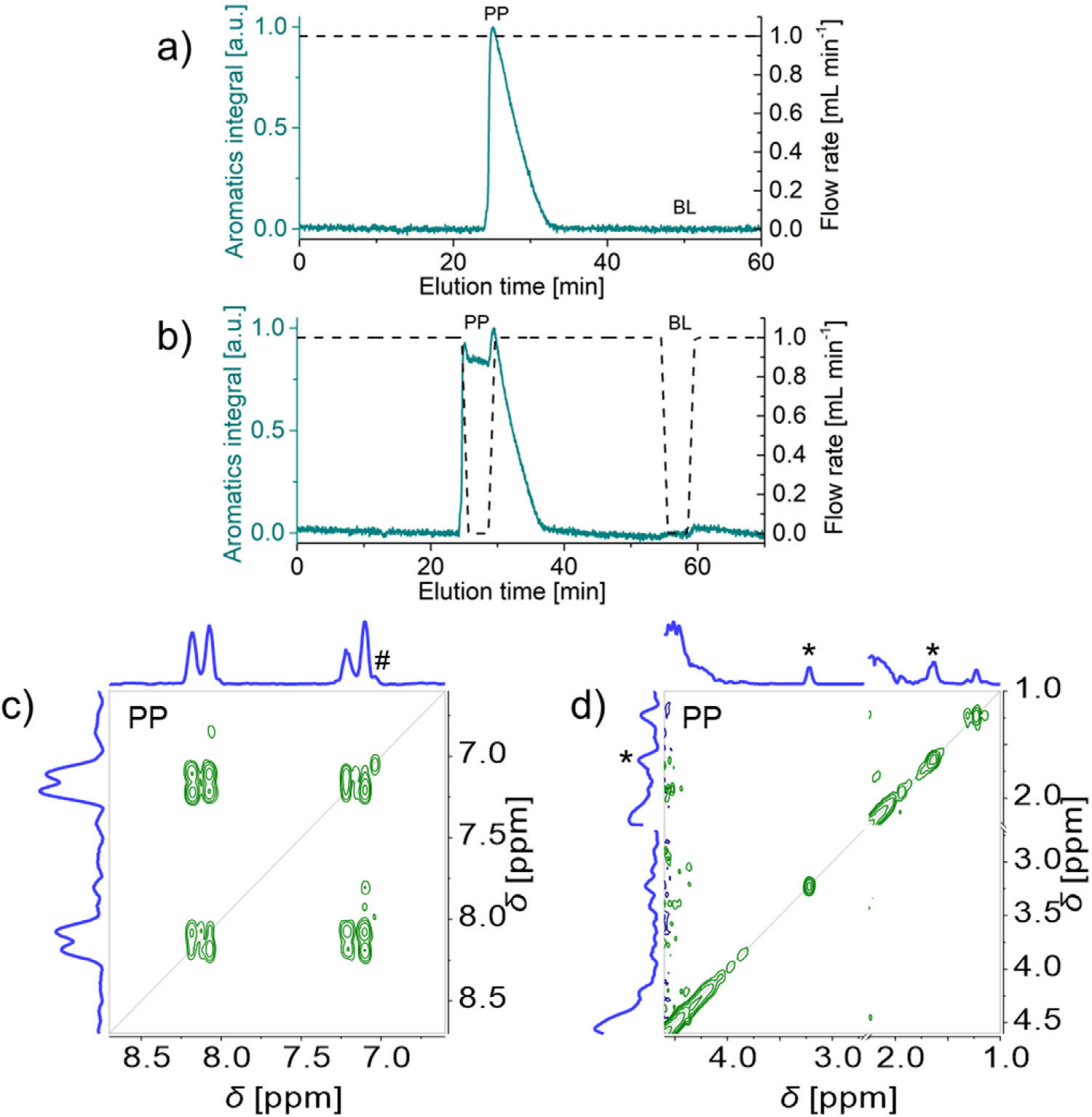
HPLC‐benchtop 2D‐NMR analysis with stop‐flow for propyl paraben PP (50 g L^−1^ in acetone/water 60/40 v/v): ^1^H NMR elugrams and corresponding flow rate profiles (a, b), COSY spectra (c, d). ^1^H NMR elugrams and corresponding flow rate profiles at constant flow rate (a) were used to determine where to stop‐flow (b), through a 1 min ramp down, a 3 min stop, and a 1 min ramp back up. COSY spectrum (at Ernst condition, with 1 scan, 256 increments in the indirect dimension, 37.5% NUS density, in 1 min 54 s) recorded during the stop in PP peak (c, d) is shown covering the aromatic region (c), and the aliphatic region (d, with acetone signal cut out for display through an axis break at 2.25–2.70 ppm). See Figure S14a,b (Supporting Information) for COSY spectrum recorded in the baseline BL. * indicates an acetone signal ^13^C satellite and # an intermodulation artifact.

The feasibility of recording COSY spectra with benchtop NMR online after HPLC separation was explored with propyl paraben by stopping the mobile phase flow during the elution of the analyte through the NMR flow cell. An NMR elugram was first recorded at 1.0 mL min^−1^ monitoring the integral of aromatic signals in 1D ^1^H NMR spectra (**Figure** **3**a). Successive 1D ^1^H NMR spectra were recorded with a 90° pulse and a 2.2 s relaxation delay corresponding to 1.3∙*T*_1_^*^ (with *T*_1_^*^ = 1.7 s, average for aromatic signals for propyl paraben at this flow rate). The peak maximum on the NMR elugram was determined to be at 25.1 min, corresponding to 25.1 mL elution volume. The flow program was thus designed as follows: constant flow at 1.0 mL min^−1^ except for 2 stops at 25.1 (propyl paraben) and 50 mL (baseline) elution volumes, each stop being set as 3 min at 0.0 mL min^−1^ with flow ramped linearly down then up over 1 min. An NMR elugram was recorded with this flow program (Figure 3b), validating that the first stop occurs at the maximum of propyl paraben elution peak in the NMR flow cell, and the second one in the baseline. Note that the lower integral observed for the plateau during the first stop is attributed to a *T*_1_^*^ effect: *T*_1_^*^ is longer at 0.0 than at 1.0 mL min^−1^ (2.2 s vs 1.7 s for the average value of aromatic signals). As the NMR acquisition parameters were chosen based on *T*_1_^*^ = 1.7 s the signal integral at 0.0 mL min^−1^ is reduced.

The same flow program with 3 min stops at 25.1 mL (propyl paraben) and 50.0 mL (baseline) elution volumes was then carried out to record COSY spectra at these elution volumes. Individual 1D ^1^H NMR spectra were recorded immediately before and after each COSY spectrum to validate the timing of the COSY spectrum. The COSY spectrum recorded in the propyl paraben peak exhibited the expected aromatic signals and splitting pattern (Figure 3c) as well as the expected methyl signal at 1.2 ppm (Figure 3d). Note that other alkyl side chain groups or their cross‐peaks were not detected. The COSY spectrum recorded in the baseline exhibited no analyte signal, only solvent signals (Figure S14a,b, Supporting Information). The ^13^C satellites of the acetone signal (identified with *) and an intermodulation artifact (identified with # and due to the presence of 2 strong solvent signals), did not interfere with these observations. The online coupling of HPLC and benchtop 2D‐NMR was thus achieved with propyl paraben stopping the flow for 3 min to record COSY spectra and sufficient NMR system stability.

The same experiment was conducted with a shorter COSY experiment, reducing the number of increments in the indirect dimension from 256 to 128 and the COSY duration from 2 to 1 min, and thus reducing the set stop time at 0.0 mL min^−1^ from 3 to 2 min (Figure S12, Supporting Information). The COSY spectrum of propyl paraben was distorted with changed relative intensities of the signals in the direct dimension and a loss of the splitting pattern in the indirect dimension. The gain in overall experimental time of only 2 min was deemed of minimal value in view of the compromised data resolution and distorted peak shapes. This approach was not pursued, and subsequent COSY spectra were all recorded again in 2 min with 256 steps in the indirect dimension.

HPLC‐benchtop 2D‐NMR was established with stop‐flow for propyl paraben. HPLC is however only relevant for the analysis of mixtures. HPLC‐benchtop 2D‐NMR with stop‐flow was thus explored for a mixture of parabens.

#### 2.3.2 Separation of Parabens

**Figure 4:**
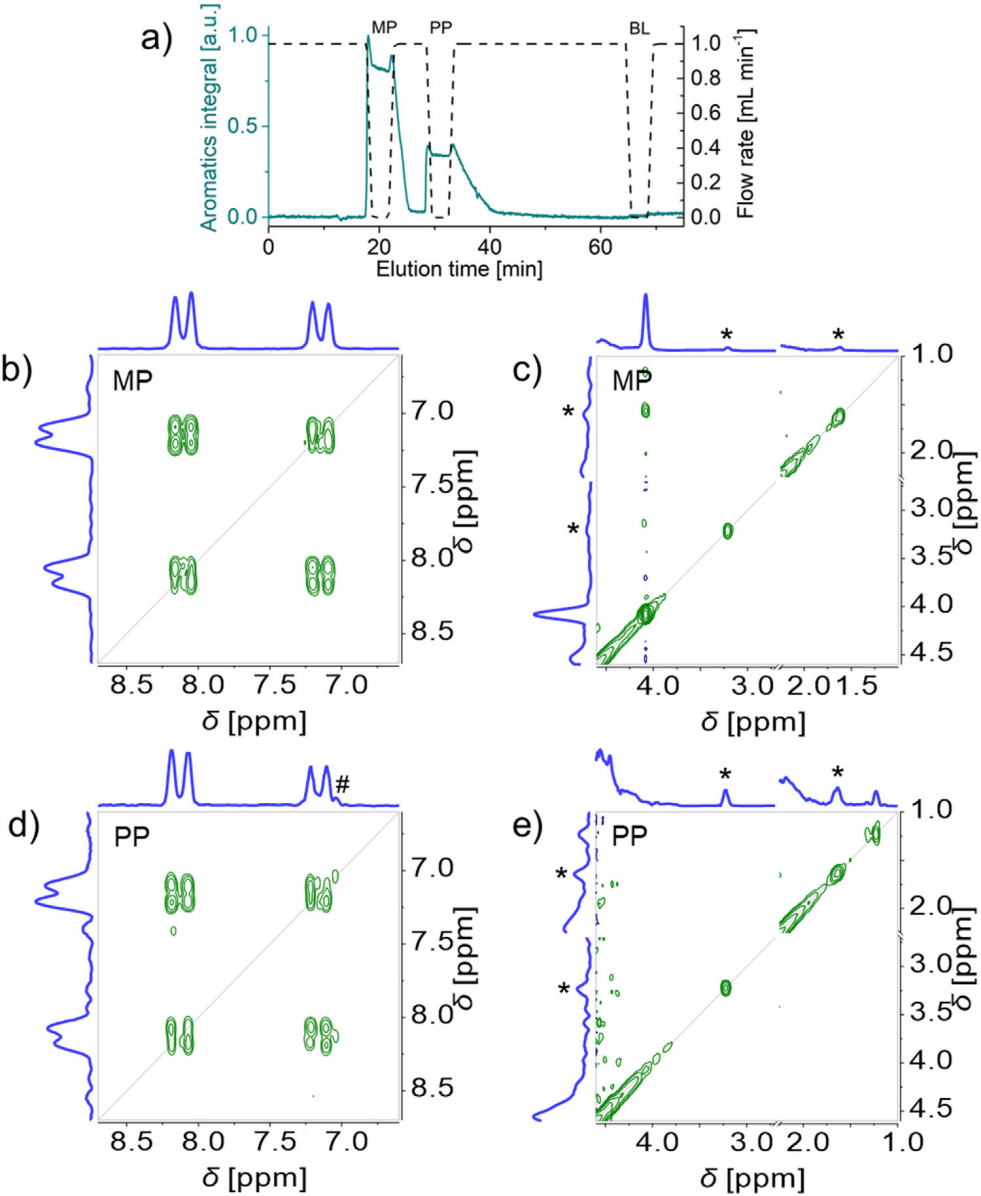
HPLC‐benchtop 2D‐NMR analysis with stop‐flow for a mixture of methyl paraben MP and propyl paraben PP (50 g L^−1^ each in acetone/water 60/40 v/v): ^1^H NMR elugram and corresponding flow rate profile (a), COSY spectra (b–e). The ^1^H NMR elugrams and corresponding flow rate profile (a) show the stop‐flow increments in the indirect dimension with a 1 min ramp down, a 3 min stop, and 1 min ramp back up. COSY spectra (at Ernst condition, with 1 scan, 256 increments in the indirect dimension, 37.5% NUS density) recorded in 1 min 54 s during the stops in MP peak (b, c) and PP peak (d, e) are shown covering the aromatic region (b, d), and the aliphatic region (c, e, with acetone signal cut out for display through an axis break at 2.25–2.70 ppm). See Figure S14c,d (Supporting Information) for COSY spectrum recorded in the baseline BL. * indicates an acetone signal ^13^C satellite and # an intermodulation artifact.

NMR elugrams were recorded at 1.0 mL min^−1^ for a mixture of methyl and propyl parabens, without and then with 3 min stops. The positions for the 3 min stops were thus validated at elution volumes of 18.2 (methyl paraben), 25.0 (propyl paraben), and 57.0 mL (baseline) (see **Figure** **4**a; Figure S13a–c, Supporting Information). Introducing the stops did not significantly affect the elution volume of the analytes. Only a slight broadening of the second analyte peaks was observed with the introduction of the stops (see Figure S13d, Supporting Information for an overlay of NMR elugrams with and without stops).

A flow program with 3 min stops at elution volumes of 18.2 mL (methyl paraben), 25.0 mL (propyl paraben), and 57.0 mL (baseline) was then carried out to record COSY spectra. Individual 1D ^1^H NMR spectra were recorded immediately before and after each COSY spectrum to validate the timing of the COSY spectrum. The COSY spectra recorded in the methyl and propyl paraben peaks exhibited the expected aromatic signals and splitting pattern (Figure 4b,d, respectively) as well as the expected methyl signals at 4.1 and 1.2 ppm (Figure 4c,e, respectively). The COSY spectrum recorded in the baseline exhibited no analyte signal, only solvent signals (Figure S14c,d, Supporting Information). The ^13^C satellites of the acetone signal and an intermodulation artifact did not interfere with these observations. The online coupling of benchtop 2D‐NMR to HPLC with stop‐flow thus allows the separation and characterization of parabens.

### 2.4 Online Coupling of HPLC and 2D‐NMR with Slow‐Flow

**Figure 5:**
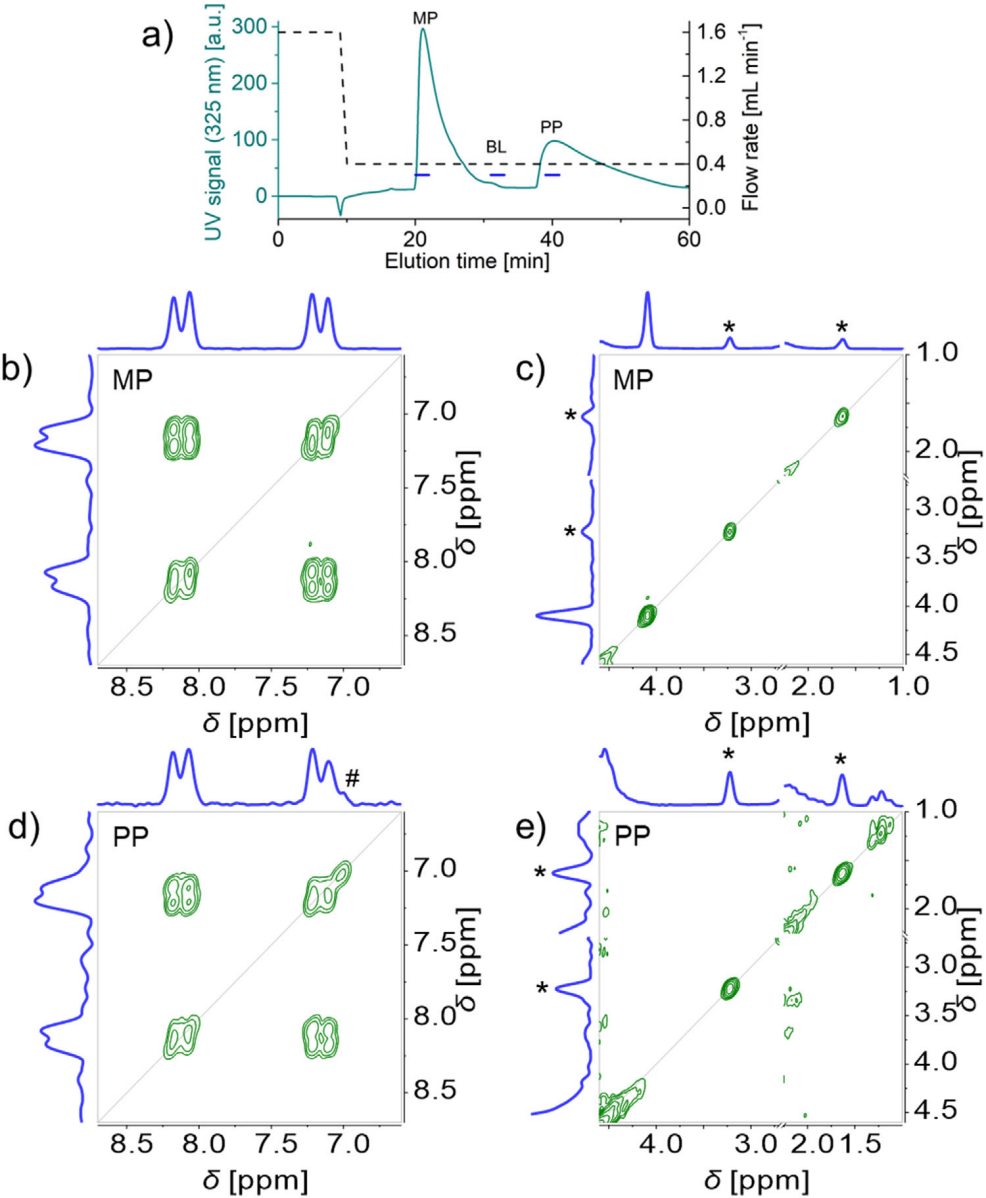
HPLC‐benchtop 2D‐NMR analysis with slow‐flow for a mixture of methyl paraben MP and propyl paraben PP (50 g L^−1^ each in acetone/water 60/40 v/v): UV absorption (325 nm) elugram and corresponding flow rate profile (a), COSY spectra (b–e). The UV absorption elugram and corresponding flow rate profile (a) show the position of the COSY spectra recording (blue lines). COSY spectra (at Ernst condition, with 1 scan, 256 increments in the indirect dimension, 37.5% NUS density) were recorded in 1 min 54 s at slow‐flow (0.4 mL min^−1^) in MP peak (b, c) and in PP peak (d, e). COSY spectra are shown covering the aromatic region (b, d), and the aliphatic region (c, e, with acetone signal cut out for display through an axis break at 2.25–2.70 ppm). See Figure S15c,d (Supporting Information) for COSY spectrum recorded in the baseline BL. * indicates an acetone signal ^13^C satellite and # an intermodulation artifact.

Another option was then explored for HPLC‐benchtop 2D‐NMR. The flow was slowed down over the whole range of the elution of the analytes in the NMR flow cell to broaden the chromatographic peaks on the elution time scale. The separation of methyl and propyl paraben was thus carried out at 1.6 mL min^−1^, except for the 15–42 mL elution volume for which the flow rate was lowered to 0.4 mL min^−1^ (ramping down over 1 min, ramping up over 2 min). The 1.6 mL min^−1^ flow rate corresponds to the maximal separation for MP according to van Deemter curve for the used column. The start of the analyte elution volume range was chosen at 15 mL just after the system peak as for different samples analytes may start eluting from there. A UV elugram (**Figure** **5**a) shows the timing of COSY spectrum acquisitions at 20.0, 31.0, and 39.0 min. Individual 1D ^1^H NMR spectra were recorded immediately before and after each COSY spectrum to validate the timing of the COSY spectrum. NMR elugrams were not used to determine the timing of COSY spectra acquisition under slow‐flow since they suffered from magnetic field drift after the flow rate change (Figure S15a,b, Supporting Information).

The COSY spectra recorded with the slow‐flow mode in the propyl and methyl paraben peaks exhibited the expected aromatic signals and splitting pattern (Figure 5b,d, respectively) as well as the expected methyl signals at 4.1 and 1.2 ppm (Figure 5c,e, respectively). The COSY spectrum recorded in the baseline exhibited no analyte signal, only solvent signals (Figure S15c,d, Supporting Information). The ^13^C satellites of the acetone signal and an intermodulation artifact did not interfere with these observations. The online coupling of benchtop 2D‐NMR to HPLC is thus also possible with slow‐flow for the separation and characterization of parabens.

### 2.5 Online Coupling of HPLC and 2D‐NMR at Steady‐Flow

**Figure 6:**
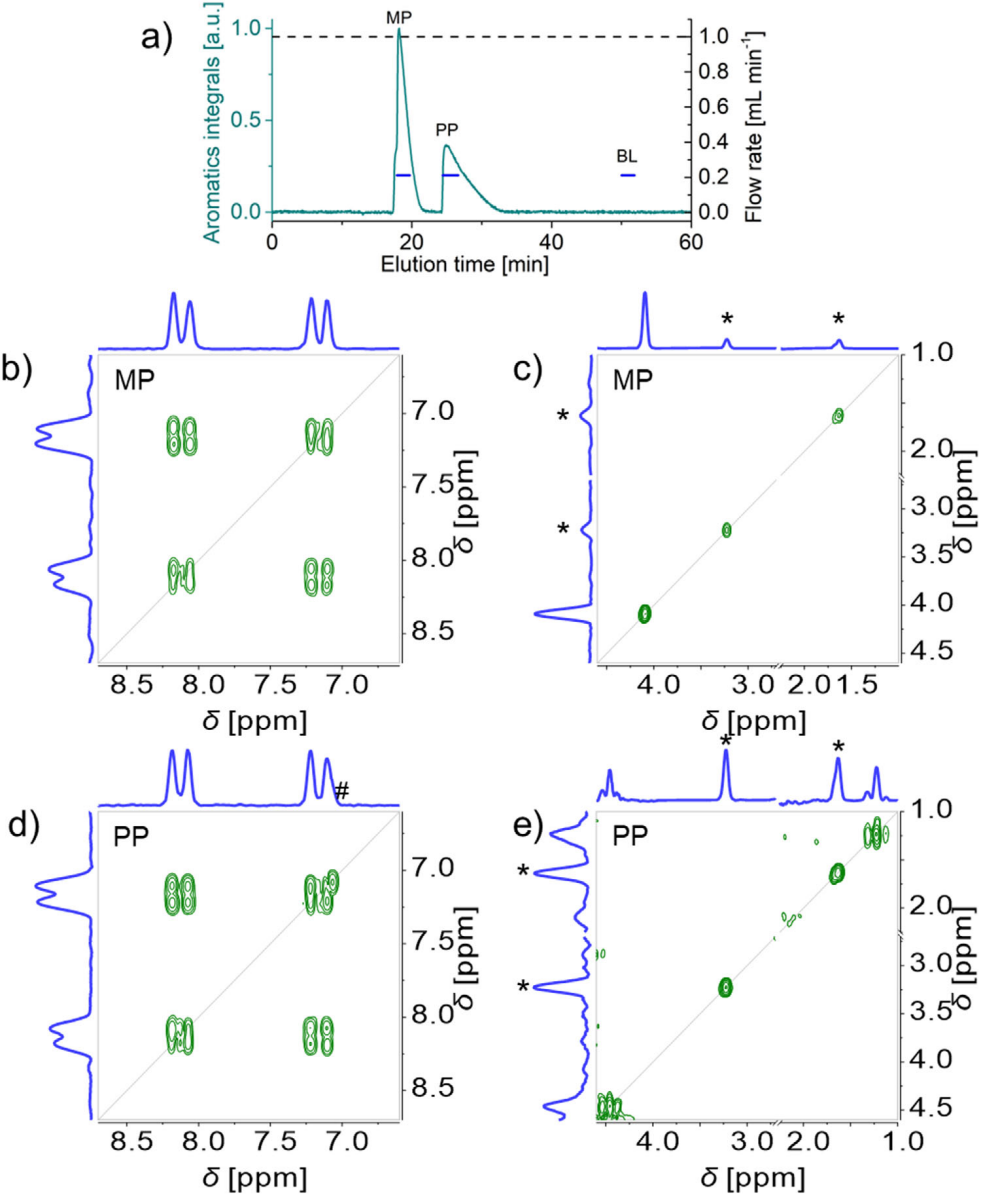
HPLC‐benchtop 2D‐NMR analysis in steady‐flow mode for a mixture of methyl paraben MP and propyl paraben PP (50 g L^−1^ each in acetone/water 60/40 v/v): ^1^H NMR elugram and corresponding flow rate profile (a), COSY spectra (b–e). The ^1^H NMR elugram and corresponding flow rate profile (a) show the position of the COSY spectra recording (blue lines). COSY spectra (at Ernst condition, with 1 scan, 256 increments in the indirect dimension, 37.5% NUS density) recorded in 1 min 54 s during the MP peak (b, c) and PP peak (d, e) are shown covering the aromatic region (b, d), and the aliphatic region (c, e, with acetone signal cut out for display through an axis break at 2.25–2.70 ppm). See Figure S16 (Supporting Information) for COSY spectrum recorded in the baseline BL. * indicates an acetone signal ^13^C satellite and # an intermodulation artifact.

Based on an observation that the chromatographic peaks of methyl and propyl parabens were at least 2 min broad at their base at 1.0 mL min^−1^, it was attempted to record COSY spectra at steady‐flow during their chromatographic separation at the constant flow rate of 1.0 mL min^−1^. The start times for COSY spectra were chosen at the half maximum of peak height on the peak increase side in the NMR elugram for methyl paraben (17.9 min corresponding to 17.9 mL elution volume) and propyl paraben (24.4 min corresponding to 24.4 mL elution volume) and at 50.0 min (corresponding to 50.0 mL elution time) for the baseline (**Figure** **6**a). The right positioning was validated through 1D ^1^H NMR spectra recorded immediately before and after each COSY spectrum.

The COSY spectra recorded in the propyl and methyl paraben peaks exhibited the expected aromatic signals and splitting pattern (Figure 6b,d, respectively) as well as the expected methyl signals at 4.1 and 1.2 ppm (Figure 6c,e, respectively). The COSY spectrum recorded in the baseline exhibited no analyte signal, only solvent signals (Figure S16, Supporting Information). The ^13^C satellites of the acetone signal and an intermodulation artifact did not interfere with these observations. The online coupling of benchtop 2D‐NMR to HPLC is thus possible even on flow at steady‐flow for the separation and characterization of parabens.

### 2.6 Discussion

Several options were successfully explored in this work for the online coupling of benchtop 2D‐NMR to HPLC through the separation and characterization of methyl and propyl parabens with COSY. These three modes are stop‐flow (with 3 min stops at the elution volumes of the analytes and in the baseline), slow‐flow (with a lower flow rate over the elution range of all analytes than for the rest of the separation) and steady‐flow (with a constant flow rate throughout the whole experiment). Their advantages and drawbacks are summarized in **Table** **1**.

**Table 1:** Advantages (+) and drawbacks (−) of the three options of HPLC‐benchtop 2D‐NMR for the separation of parabens.

Online coupling mode	Easiness to set up	Adjustability of NMR experiment duration	Analyte concentration steadiness during NMR	NMR instrument stability	NMR experiment sensitivity
Stop‐flow	−	+ +	+ +	+	++
Slow‐flow	−	−	−	−	+
Steady‐flow	+ +	− −	− −	++	−

The steady‐flow mode is the most straightforward to establish as no flow rate changes are required throughout the experiment. It has the added advantage of not exposing the column packing material to pressure changes during flow rate changes and multiple stops needed in the other options. The stop‐flow mode is the only one that is fully adjustable in terms of the NMR experiment duration, as the stop time can in principle be adjusted at will to accommodate for longer NMR experiments. In the other modes the NMR experiment duration is limited by the broadness of the chromatographic peak. While the flow rate can be decreased in the slow‐flow mode to broaden the chromatographic peaks on the elution time scale, this makes the overall HPLC‐NMR experiment much longer and very low flow rates will result in spectral instabilities, practically limiting the accessible range of NMR experiment durations. The steady‐flow mode does not offer the possibility to increase the 2D‐NMR experiment duration, unless the constant flow rate is decreased for the whole HPLC‐NMR experiment, making it excessively long. A further publication (from our team) will consider in more detail the setting up of such flow schemes and their impact on chromatographic quality.

**Figure 7:**
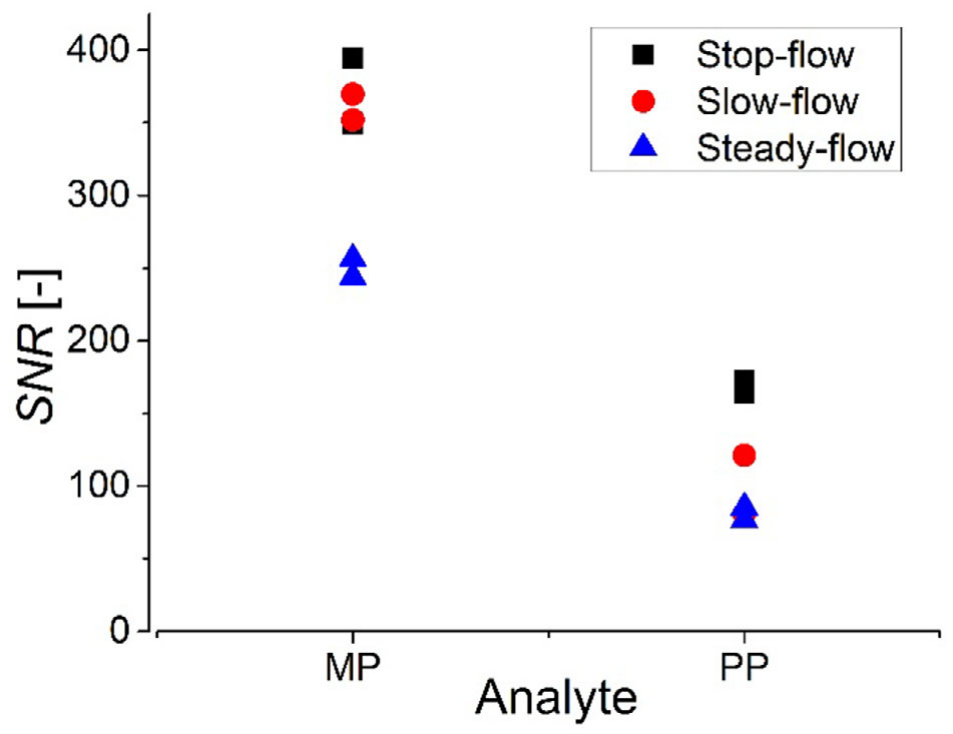
Signal‐to‐noise ratio for the cross‐peak at 8.05,7.20 ppm in the COSY spectra of methyl paraben (MP) and propyl paraben (PP) recorded with HPLC‐benchtop 2D‐NMR in stop‐flow, slow‐flow, and steady‐flow modes. See Figures 4, 5, 6 and experimental section for experimental conditions (including: semi‐preparative C18 column, 1 mL injection volume, 50 g L^−1^ of each paraben).

Only with the stop‐flow mode is the analyte concentration constant over the COSY experiment duration (for different increments in the indirect dimension). In both the slow‐flow and steady‐flow modes the COSY spectrum is recorded while the analyte is flowing in the NMR flow cell, with different concentrations for different increments recorded along the chromatographic peak. This could lead to distortions in NMR line shapes, which were not observed in this work. NMR instrument stability is critical to the quality of the data, as the chemical shifts scale in parts per million. Some instabilities were observed in the NMR elugram of the slow‐flow experiment, which were attributed to a slight magnetic field drift after the flow rate change due to a slight temperature change of the solution entering the NMR flow cell active volume after exiting the HPLC column. They were negligible in the stop‐flow mode because of the short stops (3 min). The steady‐flow mode is overall less sensitive than the other modes, while the stop‐flow mode exhibits a similar sensitivity for the methyl paraben peak and a higher sensitivity for the propyl paraben peak compared to the slow‐flow mode (**Figure** **7**; see Table S1, Supporting Information for values).

Overall, the steady‐flow and stop‐flow options both have strong advantages, namely the easiness to set up and NMR instrument stability for the steady‐flow mode, the adjustability of NMR experiment duration, the analyte concentration steadiness during NMR and NMR instrument sensitivity for the stop‐flow mode. Both can be recommended for future HPLC‐benchtop 2D‐NMR experiments. It is advised to use the steady‐flow mode when analyte concentrations and injection volumes are sufficient for the chromatographic peaks to be broad enough (without compromising the chromatographic resolution), and the stop‐flow mode in other cases.

Compared to the previous HPLC‐NMR(2D) carried out at high field, the HPLC‐benchtop 2D‐NMR established in this work presents both advantages and drawbacks. On the one hand, benchtop NMR spectrometers offer the big advantage of a smaller footprint and lower costs, especially in terms of maintenance and running costs, as no cryogenic liquids or deuterated solvents are necessary or have been used.^[^ ^18^ ^]^ This method also suffers from a lower sensitivity due to the lower magnetic field, which made a sensitivity enhancement necessary, as shown in the present work. As a consequence, rapid 2D‐NMR techniques such as the ultrafast, single scan 2D‐NMR spectroscopy^[^ ^28^ ^]^ which require strong sensitivity have been implemented mostly at high field. Their implementation on benchtop NMR spectrometers for the analysis of mixtures under flow is currently limited to reaction monitoring, and there are very few reports of its hyphenation to HPLC,^[^ ^29^, ^30^ ^]^ all with high field NMR spectrometers.^[^ ^40^ ^]^ Another rapid 2D‐NMR experiment family based on Hadamard encoding^[^ ^32^ ^]^ was only reported once to be hyphenated to HPLC, on a high‐field NMR spectrometer.^[^ ^33^ ^]^ Despite longer 2D‐NMR experiment durations (2 min compared to previously reported 12–37 s at 500 or 600 MHz^[^ ^29^, ^30^, ^33^ ^]^), the HPLC‐NMR(2D) was successfully implemented here on a benchtop NMR instrument at 80 MHz.

## 3 Conclusion

The online coupling of 2D‐NMR to HPLC was successfully established for the first time with a benchtop NMR instrument (at 80 MHz). It was demonstrated on a mixture of parabens, additives commonly used in cosmetics, with COSY (^1^H‐^1^H COrrelation SpectroscopY) spectra. It took advantage of three different options: i) stop‐flow (with short stops at the analyte peaks), ii) slow‐flow (with a lower, constant flow rate for the whole elution range of analytes) and iii) steady‐flow (at a constant flow rate throughout the experiment). It was made possible through the improvement of the COSY experiment aiming at reducing the experiment duration for the 2D‐NMR spectra. The repetition delay and nutation angle were reduced and non‐uniform sampling (NUS) implemented. This reduced the experiment duration from about 1 h down to 2 min without compromising the sensitivity as assessed through the signal‐to‐noise ratio per square root of experiment duration. HPLC‐benchtop 2D‐NMR provides more in‐depth chemical information on the separated analytes compared to HPLC‐benchtop 1D‐NMR, with a smaller footprint and at lower costs compared to HPLC‐high‐field 2D‐NMR. The steady‐flow mode is recommended for concentrated analytes and broad peak profiles, the stop‐flow mode in other cases.

Further work is in progress to extend this methodology to other experiments (e.g., Heteronuclear Single‐Quantum Correlation, HSQC,^[^ ^41^, ^42^ ^]^ to provide ^1^H‐^13^C correlation information) and to more analytes (such as polymers with or without small molar mass additives). Gradient elution, commonly used in HPLC, could be implemented to narrow chromatographic peaks thereby increasing their height and increasing the sensitivity in the NMR dimension in the stop‐flow mode. A solvent suppression scheme such as WET (Water suppression Enhanced through *T*_1_ effects, first developed at high field^[^ ^43^ ^]^ and also implemented on benchtop spectrometers^[^ ^44^ ^]^) could be introduced in the COSY experiment to comparatively increase the sensitivity for non‐solvent signals. This will give access to lower analyte concentration ranges for an in‐depth characterization via the HPLC‐benchtop 2D‐NMR hyphenation.

## 4 Experimental Section

### Materials

Methyl paraben (methyl‐4‐hydroxybenzoate, 99%) was sourced from Thermo Scientific (Kandel, Germany), propyl paraben (propyl‐4‐hydroxybenzoate, 99+%) from ACROS (Geel, Belgium), acetone (≥99.8%, HPLC grade) and water (HPLC gradient grade) from Fisher Scientific (Schwerte, Germany). See Figure 1b and Figure S1 (Supporting Information) for molecular structures.

### Setup for online HPLC‐benchtop NMR hyphenation

The HPLC‐NMR (80 MHz) setup (Figure S2, Supporting Information) is described in previous work.^[^ ^12^ ^]^ The HPLC instrument (Agilent Technologies, Waldbronn, Germany, unless otherwise specified) was composed of a quaternary pump (1260 Infinity II) with an in‐line degasser, a manual injection valve (Rheodyne, 7725i) with a stainless steel injection loop (1000 µL), a semi preparative C18 column (C18 aqua, 5 µm particle size, 125 Å pore size, 250 × 10 mm i.d., Phenomenex, Torrance, USA), a thermostatted column compartment (TCC 6500, PSS, Mainz, Germany), a UV detector (1260 Infinity Variable Wavelength Detector), a differential refractive index (DRI) detector (1260 Infinity). WinGPC software (version 8.32, build 8844, Agilent Technologies, Waldbronn, Germany) was used for pump, UV and DRI control, as well as UV and DRI data acquisition.

An 80 MHz NMR spectrometer (^1^H single channel, Magritek, Aachen, Germany), equipped with an external ^19^F lock, was connected between the column and the UV detector. A custom‐made cone shaped NMR glass flow cell was used with the following specifications: 30° cone angle, active length of 15 mm (for online coupling, Sections 2.3–2.5) or 18 mm (for COSY experiment enhancement, Section 2.2), 4.23 mm active region internal diameter, active volume of 211 or 226 µL, total volume of 496 or 516 µL (see published specifications for flow cells FC9 and FC10 in^[^ ^11^ ^]^ and Figure S2, Supporting Information for flow cell geometry). The flow cell was connected to the column and the UV detector through 0.25 mm i.d. PTFE tubing. SpinsolveExpert (Version 1.41.18, Magritek, Aachen, Germany) was used for NMR control and NMR data acquisition. COSY spectra were processed and plotted with MestReNova (Version 14.1.2‐25024, Mestelab Research, Santiago de Compostela, Spain), series of 1D ^1^H NMR spectra were processed with in‐house Matlab scripts to determine the integral of aromatic signals as a function of elution time. Profiles (flow rate, ^1^H NMR elugrams, UV elugrams) and graphs were plotted with OriginPro (version 9.0.0, OriginLab, Northampton, MA, USA).

### COSY Experiment Enhancement

The NMR parameters were improved with the hyphenation setup described above including the flow cell FC10, with a stock solution of propyl paraben (30 g L^−1^ in acetone/water 60/40 v/v, filtered through a 0.45 µm porosity hydrophilic PTFE filter) circulating at 0.0, 0.4, or 1.0 mL min^−1^ as specified. This is referred to in this work as the circulatory setup. Unless otherwise specified, the ^1^H‐^1^H COSY spectra were recorded with the pulse sequence shown in Figure 1a using 0 dB 90° and 45° pulses, 128 or 256 increments in the indirect dimension with a 1 kHz bandwidth (13 ppm), 5 ms spoiler pulses at 6000 a.u. amplitude corresponding to about 0.012 T m^−1^, 1 ms dwell time and 512 points for acquisition, 1 scan per increment, 4 dummy scans, a NUS density of 37.5%, and different relaxation delays set to multiples of the apparent average longitudinal relaxation time *T*_1_^*^ of the aromatic signals: 5∙*T*_1_^*^ or 1.3∙*T*_1_^*^ with the standard COSY pulse sequence with two 90° pulses, ‐ln(cos(45°))∙*T*_1_^*^ (≈0.35∙*T*_1_^*^) with the COSY‐45 pulse sequence with one 90° pulse and one 45° (the latter being referred to Ernst angle conditions in this work). *T*_1_^*^ values of the aromatic signals at different flow rates are shown in Figure S3 (Supporting Information). COSY spectra were processed with zero filling to 2048 points in both dimensions, apodization with sine square 0° and first point at 0.5 in indirect dimension, apodization with sine square 0° and 1 Hz exponential line broadening in direct dimension, automatic baseline correction with 3rd order polynomial in both dimensions. COSY spectra were plotted with the sums of spectra over the displayed chemical shift range as projections. COSY spectra were calibrated with the acetone signal at 2.43 ppm.^[^ ^12^ ^]^ Signal‐to‐noise ratio *SNR* was determined in COSY spectra with noise over 9.0–10.5 ppm and signals over 6.0–8.3 ppm using MestReNova; the *SNR* values for the cross‐peak at 8.05,7.20 ppm are reported in this work.

### HPLC‐NMR Online Coupling

The hyphenation setup described above included the flow cell FC9. The sample was either propyl paraben (50 g L^−1^) or a mixture of methyl and propyl parabens (50 g L^−1^ each) in the eluent (filtered through 0.45 µm porosity hydrophilic PTFE filters). The eluent was an acetone/water mixture (60/40 v/v, manually premixed to decrease flow instabilities originating from volume contraction upon mixing). The column oven was set to the NMR magnet temperature of 26.5 °C. The UV detector was set to 325 nm. Different modes were explored for the flow rate profiles: i) online coupling with stop‐flow using flow rate at 1.0 mL min^−1^ with a few stops to record COSY spectra (ramping linearly down to 0.0 mL min^−1^ over 1 min, staying at 0.0 mL min^−1^ for 3 min, ramping linearly back up to 1.0 mL min^−1^ over 1 min); ii) online coupling with slow‐flow using flow rate at 1.6 mL min^−1^ until the system peak is eluted then slowing the flow to 0.4 mL min^−1^ over the whole elution range of parabens to record COSY spectra before accelerating back to 1.6 mL min^−1^ (linearly ramping down over 1 min and linearly ramping back up over 2 min); and iii) online coupling at a steady flow rate of 1.0 mL min^−1^ over the whole elution profile.

For ^1^H NMR elugrams consecutive 1D ^1^H NMR spectra were measured with presaturation at the water and acetone resonance frequencies for 1000 ms at ‐65 dB, a 90° pulse at 0 dB, a 500 µs dwell time and 2048 points for acquisition, 1 scan, and a 2.2 s repetition time (corresponding to 1.3∙*T*_1_^*^).

The COSY spectra were recorded using the COSY‐NUS pulse sequence, with a 90° and a 45° pulse at 0 dB, 256 increments in the indirect dimension with a 1 kHz bandwidth (13 ppm), 5 ms spoiler pulses of 6000 a.u. amplitude corresponding to about 0.012 T m^−1^, 1 ms dwell time and 512 points for acquisition, 1 scan per increment, 4 dummy scans, a NUS density of 37.5%, and a relaxation delay of ‐ln(cos(45°))∙*T*_1_^*^, in about 2 min. COSY spectra were processed and plotted as described above (COSY experiment enhancement section). To control the correct timing of COSY spectra recording, the recording of each COSY spectrum was immediately preceded and immediately followed by the recording of a 1D ^1^H NMR spectrum with a 90° pulse at 0 dB, 200 µs dwell time and 8192 points for acquisition, 1 scan, and at least 5∙*T*_1_^*^ wait before this scan.

## Conflict of Interest

The authors declare no conflict of interest.

## Supporting information



Supporting Information

## Data Availability

The data that support the findings of this study are available from the corresponding author upon reasonable request.
