# Supplementary material for: Online Coupling of 80 MHz Benchtop 2D‐COSY NMR to HPLC
Source: Macromol Rapid Commun. 2025 May 18;46(23):2500239. doi: 10.1002/marc.202500239 (PMC12687683; doi:10.1002/marc.202500239)
Supplement: Supplementary file 1 — Supporting Information [file MARC-46-2500239-s001.docx]

Supporting Information

Online Coupling of 80 MHz Benchtop 2D-COSY NMR to HPLC

Marianne Gaborieau*, Markus Matz, Johanna Tratz, Michael Pollard, Manfred Wilhelm*

# S1. Chemical structures

**
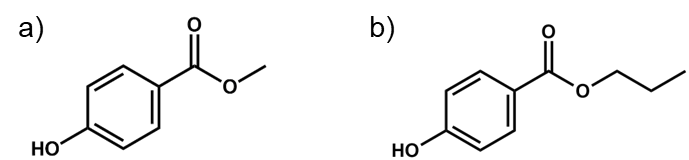
**

**Figure S1.** Chemical structures of methyl paraben (a) and propyl paraben (b).

# S2. Instrumentation


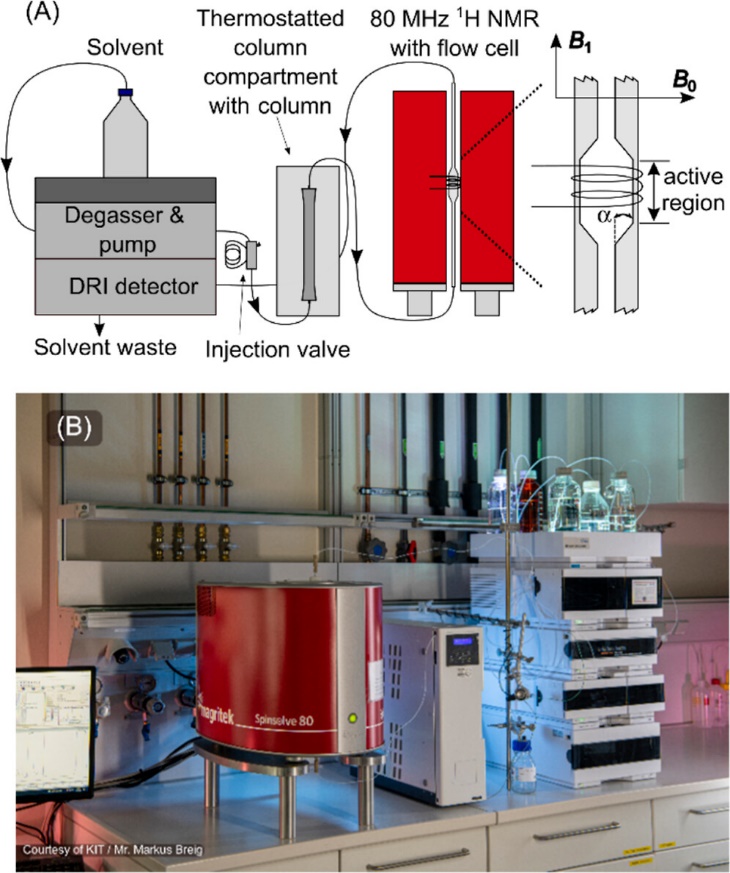


**Figure S2.** Schematic (A) and picture (B) of the online hyphenation of an 80 MHz benchtop NMR to an HPLC in LAC mode. For onflow NMR detection, a custom-made glass flow cell is used. Reprinted with permission from J. Phys. Chem. B 2024, 128, 39, 9512-9524. Copyright 2024 American Chemical Society. Photograph courtesy of Mr. Markus Breig, KIT.

# S3. Apparent relaxation times


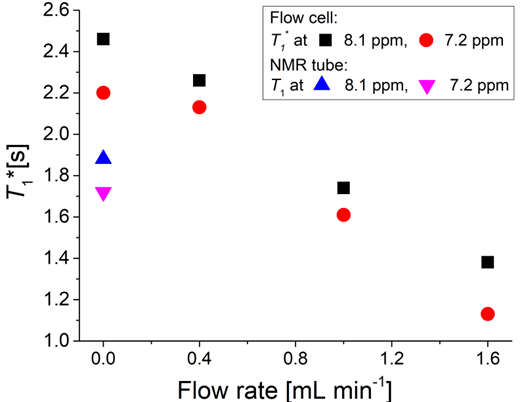


**Figure S3.** Apparent *T*_1_^*^ relaxation rates or *T*_1_ relaxation rates of aromatic ^1^H NMR signals of propyl paraben (30 g L^-1^ in acetone/water 60/40 v/v) at 80 MHz, measured in NMR tube or with the circulatory setup at different flow rates.

The (apparent) *T*_1_ relaxation times of aromatic signals of propyl paraben (30 g L^-1^ in acetone/water 60/40 v/v) were measured with the *T*_1_ inversion recovery experiment, with 10 to 40 steps from 10 ms to 4 or 5 s, 16 or 64 scans, in 1 h 24 min to 4 h 16 min (Figure S3). As expected, *T*_1_^*^ decreases with flow rate (due to the decrease in residence time in the NMR detection volume^[1]^) and increases with sample degassing (which removes oxygen).

# S4. COSY experiment enhancement

### S4.1. Spectra before, during, after enhancement

**
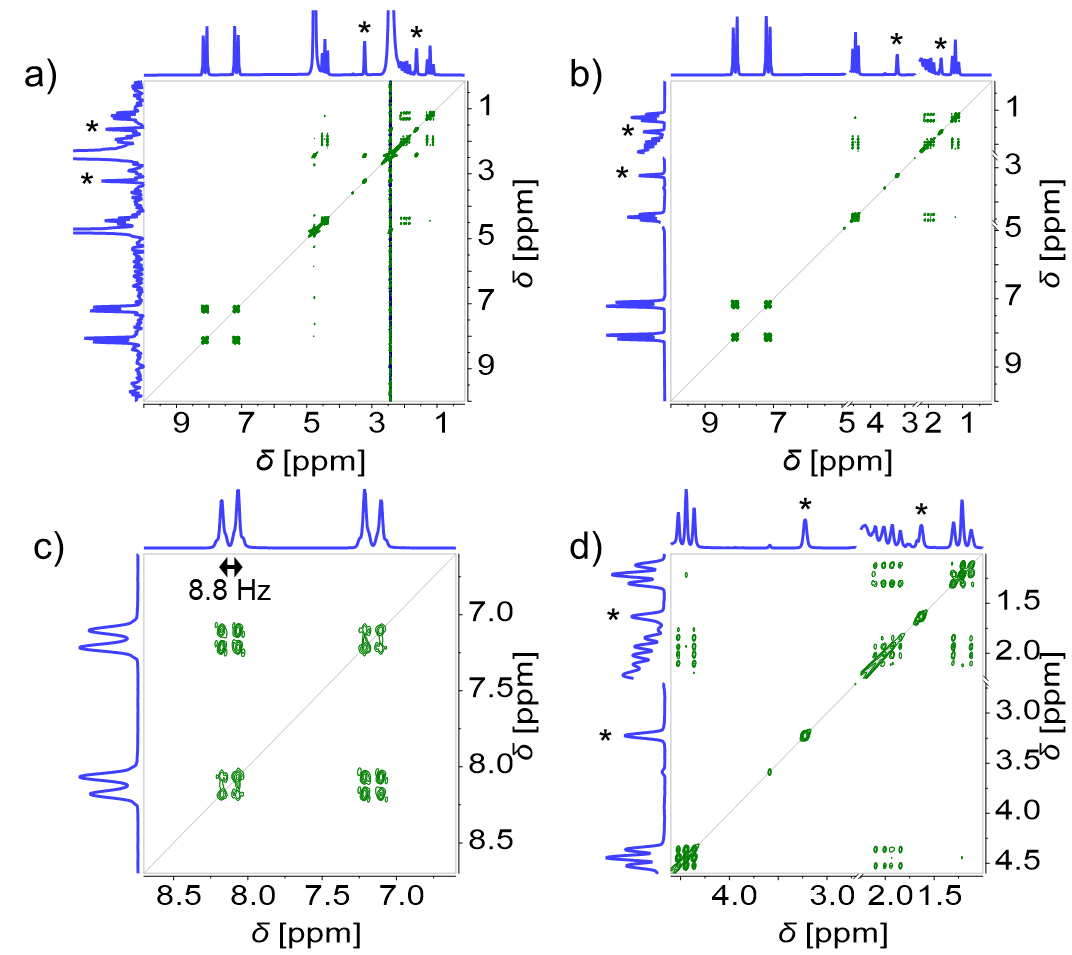
**

**Figure S4.** Reference long COSY spectrum of propyl paraben (30 g L^-1^ in acetone/water 60/40 v/v), recorded at 0.0 mL min^-1^ with the circulatory setup, with 5∙*T*_1_^*^ relaxation delay, 4 scans, 512 increments in the indirect dimension in 7 h 15 min. The COSY spectrum is shown at full 0-10 ppm scale (a), at full 0-10 ppm scale with water and acetone signal cut out for display (b, through axis breaks at 2.25-2.70 and 4.60-4.90 ppm), covering the aromatic region (c) and the aliphatic region (d, with axis break at 2.25-2.70 ppm). * indicates an acetone signal ^13^C satellite. Note that some spectra are also shown Figure 1 (a, c, d).

**
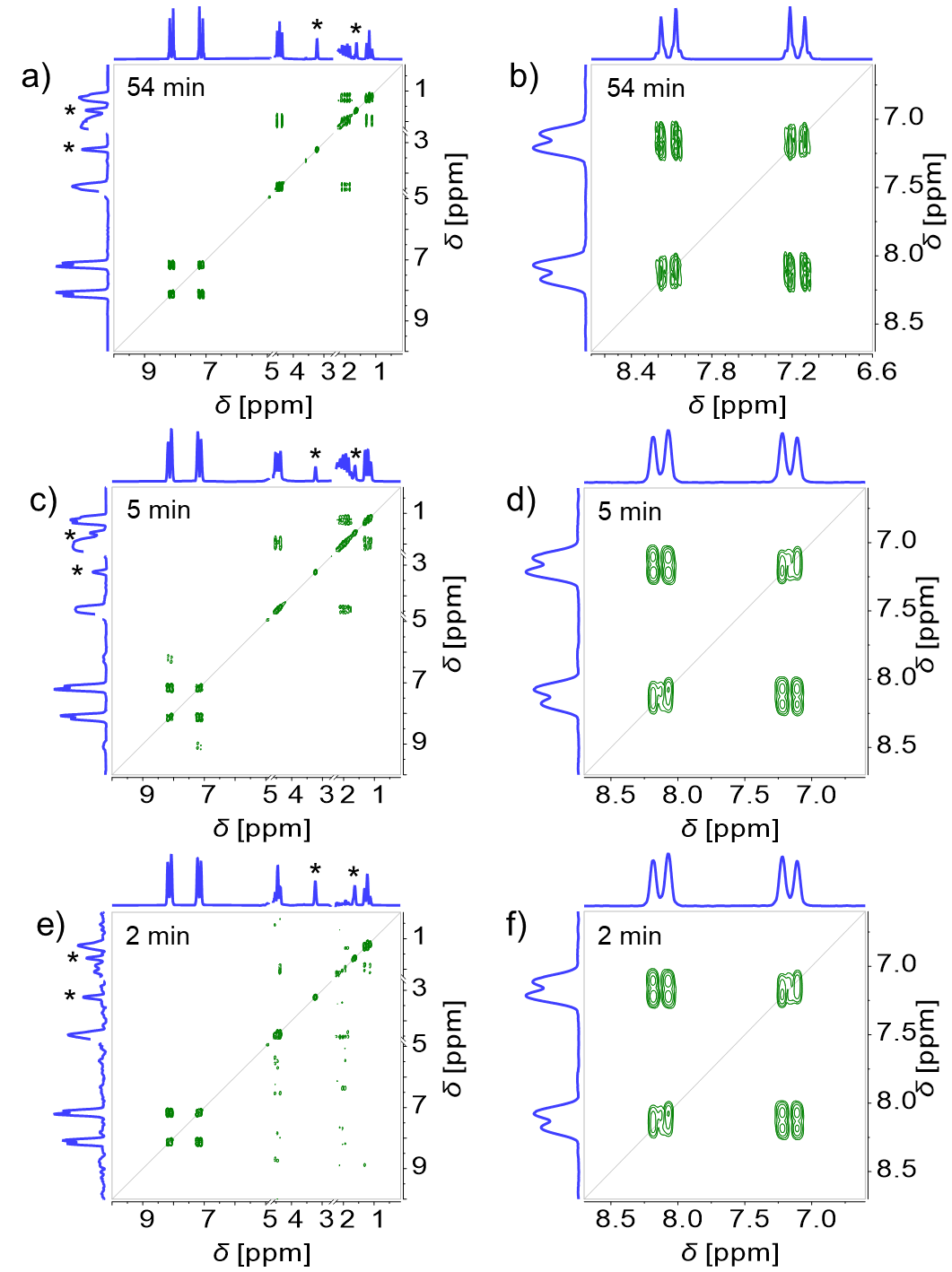
**

**Figure S5.** COSY spectra of propyl paraben (30 g L^-1^ in acetone/water 60/40 v/v) relevant for enhancement at 0.0 mL min^-1^ with the circulatory setup. Spectra were recorded before enhancement (a, b, with a 5∙*T*_1_^*^ relaxation delay, 1 scan, 256 increments in the indirect dimension, no NUS, in 54 min 51 s), after repetition delay decrease (c, d, at Ernst condition, with 1 scan, 256 increments in indirect dimension, no NUS, in 4 min 52 s), and at the end of enhancement (e,f, at Ernst condition, with 1 scan, 256 increments in indirect dimension, 37.5 % NUS density, in 1 min 54 s). Spectra are shown at full 0-10 ppm scale (a, c, e, with water and acetone signals cut out for display through axis breaks at 2.25-2.70 and 4.60-4.90 ppm) and covering the aromatic region (b, d, f). * indicates an acetone signal ^13^C satellite.

### S4.2. Enhancing sensitivity through repetition delay

**Table S1**. Flip angle, relaxation delay, COSY experiment duration and *SNR* of signal at 8.05,7.20 ppm at 0.0 mL min^-1^ with the circulatory setup for propyl paraben (30 g L^-1^ in acetone/water 60/40 v/v).

| Condition | Flip angle | Relaxation delay | Experiment duration | *SNR* |
| --- | --- | --- | --- | --- |
| Ernst | 45° | -ln(cos(*α*))∙*T*_1_^*^  = 1.1 s | 4 min 52 s | 558 ± 14 |
| 1.3∙*T*_1_^*^ | 90° | 1.3∙*T*_1_^*^  = 3.5 s | 15 min 1 s | 1934 ± 52 |
| 5∙*T*_1_^*^ | 90° | 5∙*T*_1_^*^  = 12.8 s | 54 min 21 s | 1924 ± 29 |

### S.4.3. NUS to reduce experiment duration


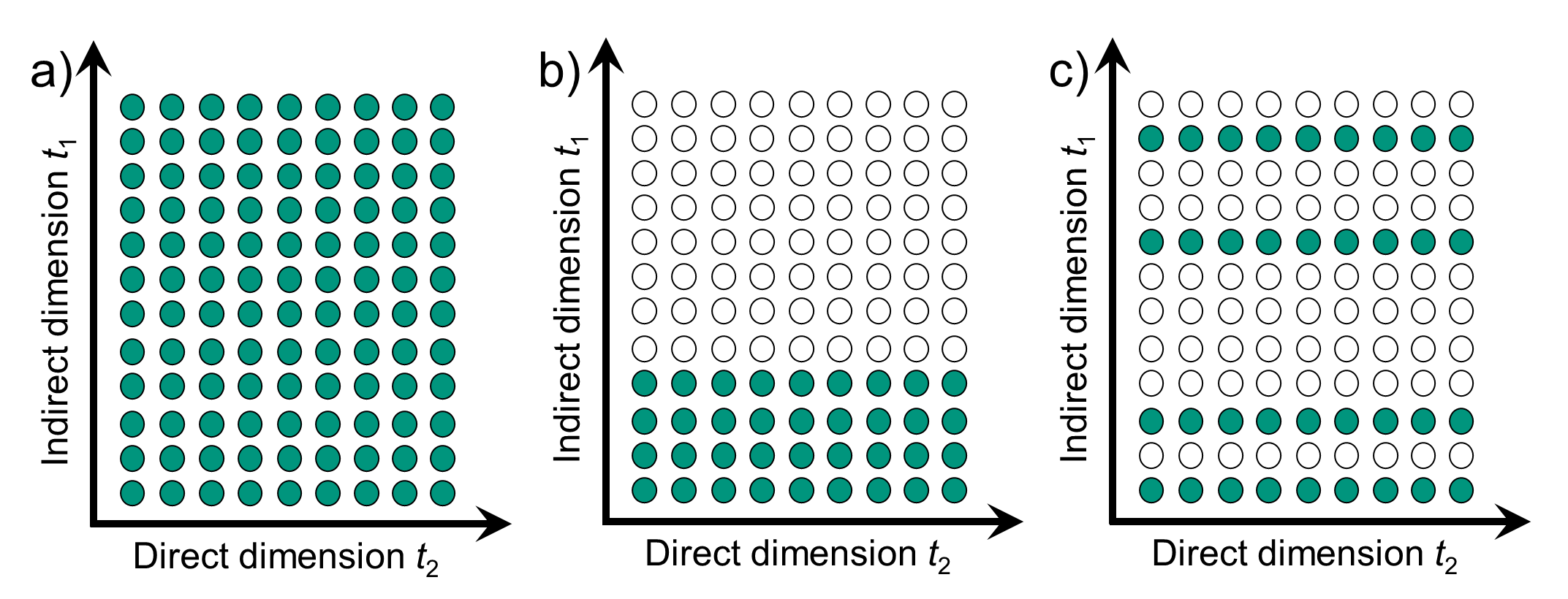


**Figure S6.** Schematic representation of non-uniform sampling (NUS): full linear sampling scheme with all increments recorded in indirect dimension *t*_1_ (a), linear sampling scheme acquiring only the first 33 % of indirect dimension (b), non-uniform sampling with random sampling at 33 % NUS density (c). Adapted from https://magritek.com/

**
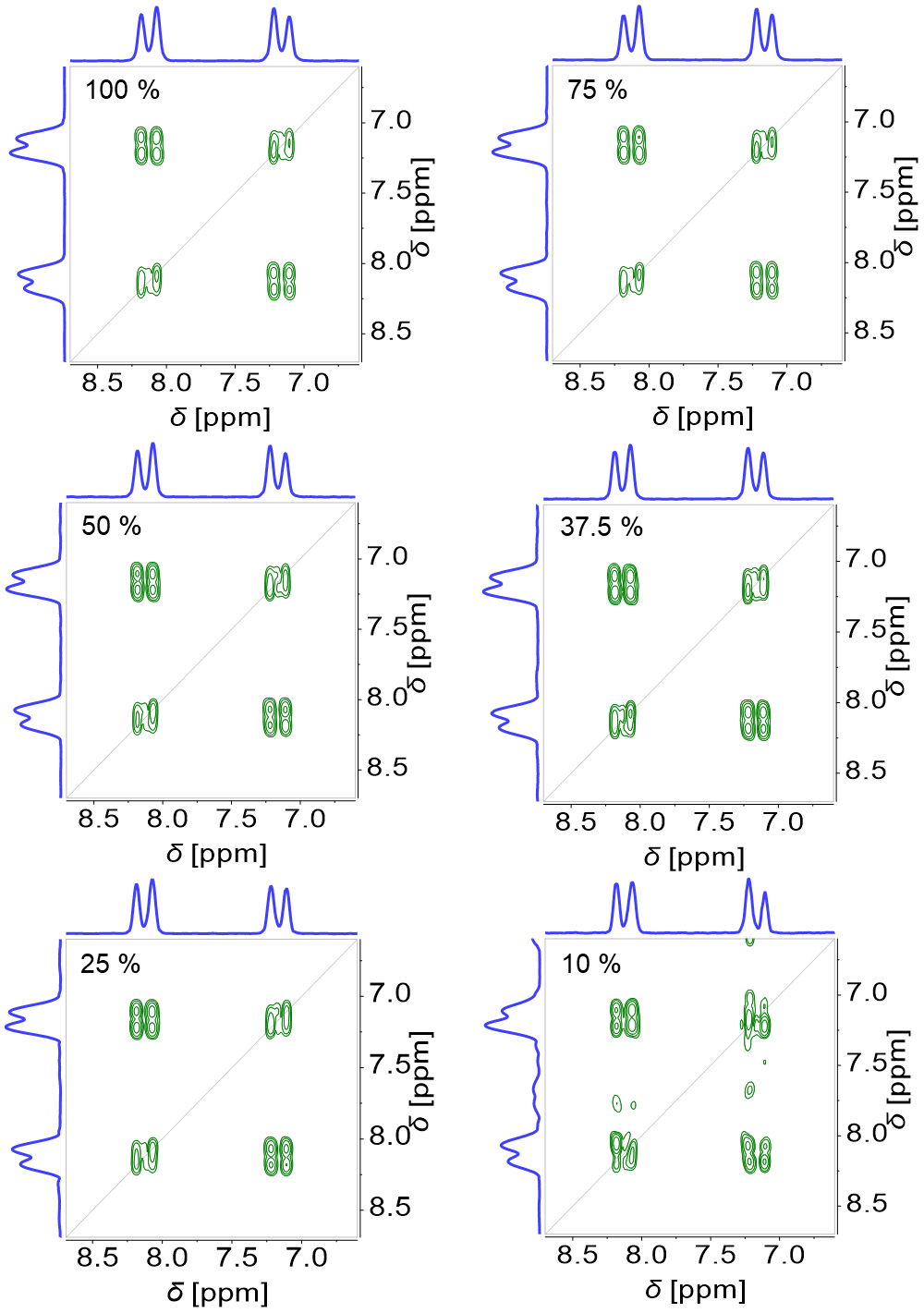
**

**Figure S7.** Aromatic region of the COSY spectrum of propyl paraben (30 g L^-1^ in acetone/water 60/40 v/v) at 0.4 mL min^-1^ with circulatory setup with 256 increments in the indirect dimension, recorded with different NUS densities.


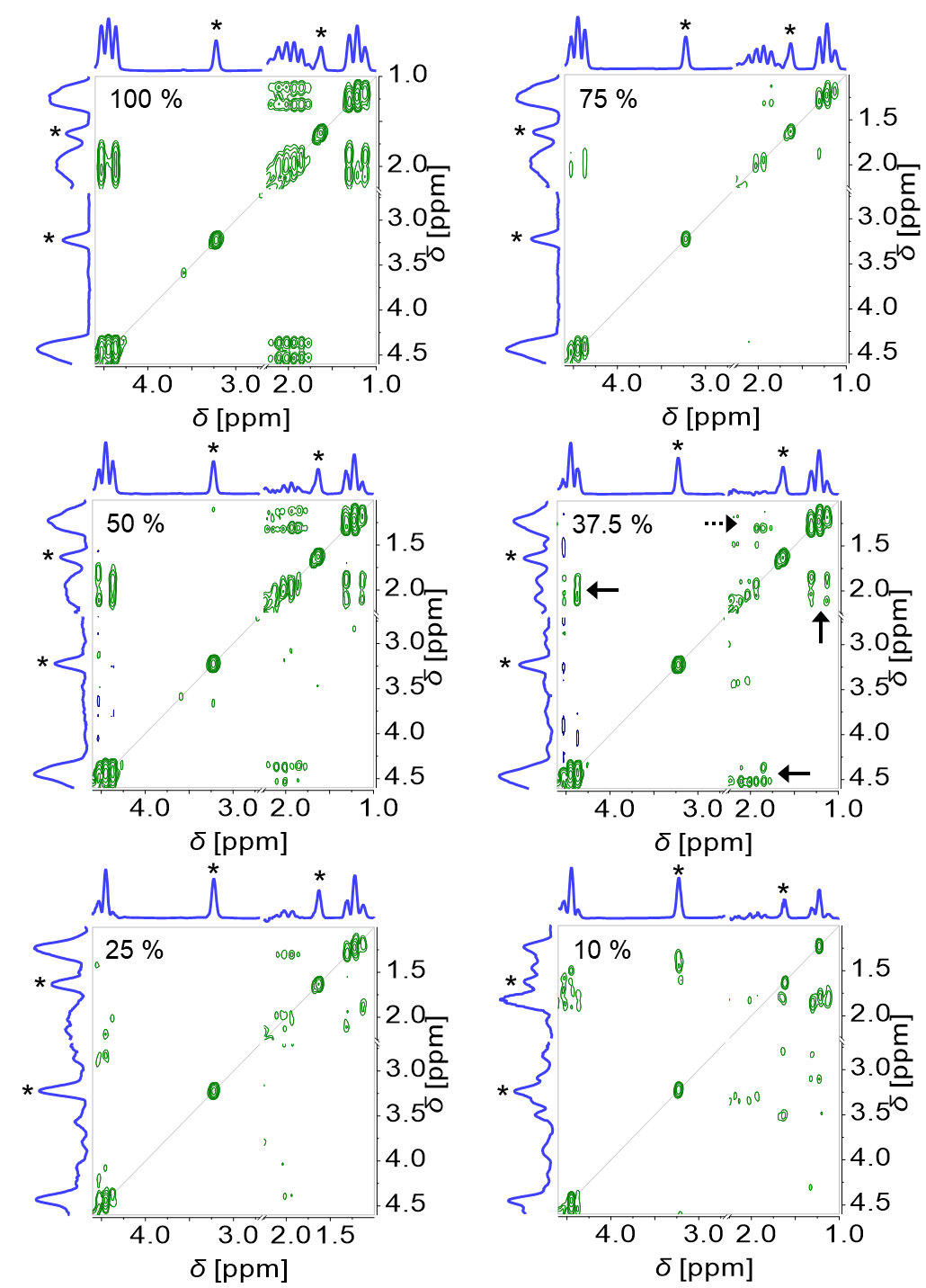


**Figure S8.** Aliphatic region of the COSY spectrum of propyl paraben (30 g L^-1^ in acetone/water 60/40 v/v) at 0.4 mL min^-1^ with circulatory setup with 256 increments in the indirect dimension, recorded with different NUS densities (with acetone signal cut out for display through an axis break at 2.25-2.70 ppm). * indicates an acetone signal ^13^C satellite. Arrows indicate cross peaks of the alkyl side groups.


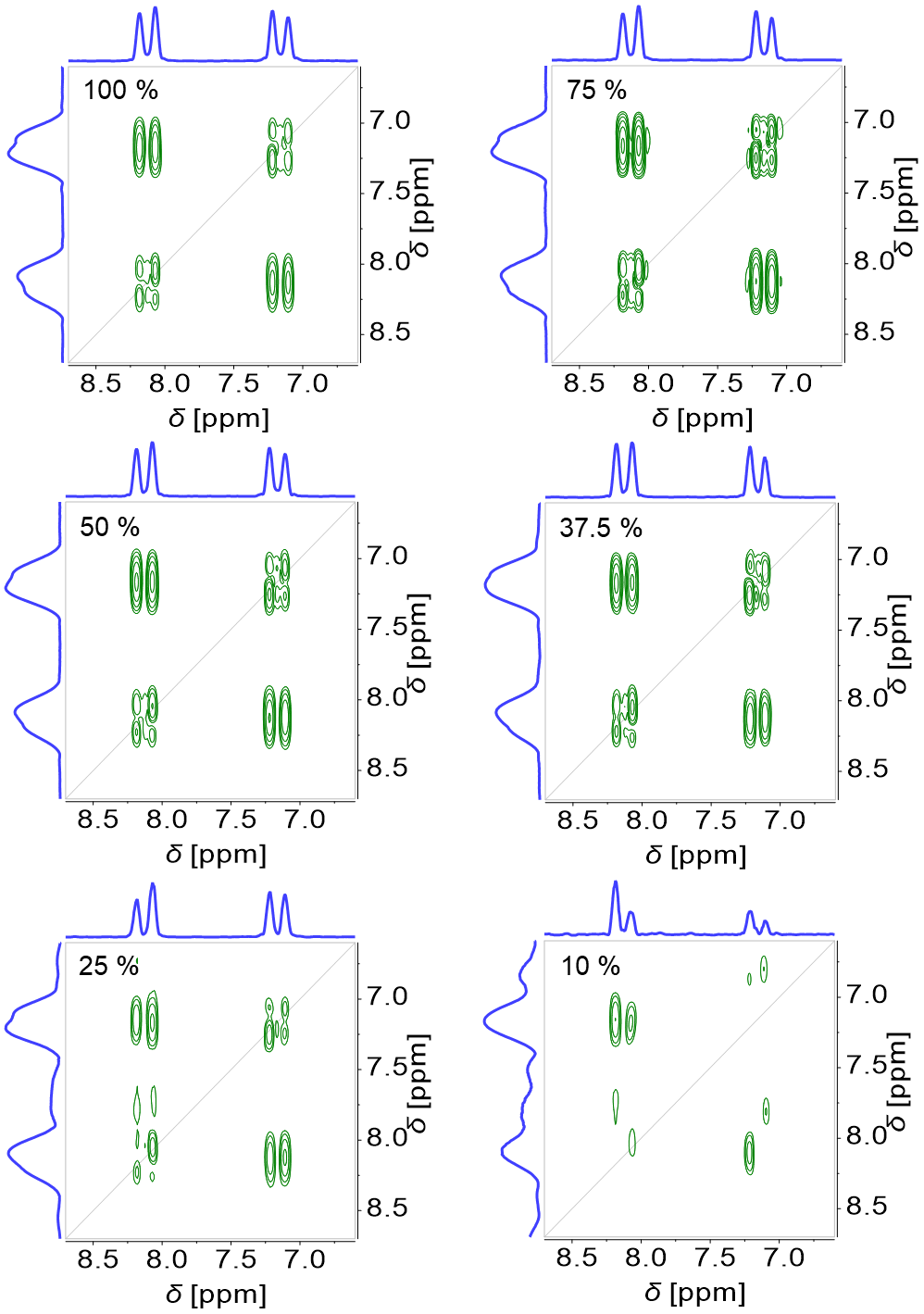


**Figure S9.** Aromatic region of the COSY spectrum of propyl paraben (30 g L^-1^ in acetone/water 60/40 v/v) at 0.4 mL min^-1^ with circulatory setup with 128 increments in the indirect dimension, recorded with different NUS densities.


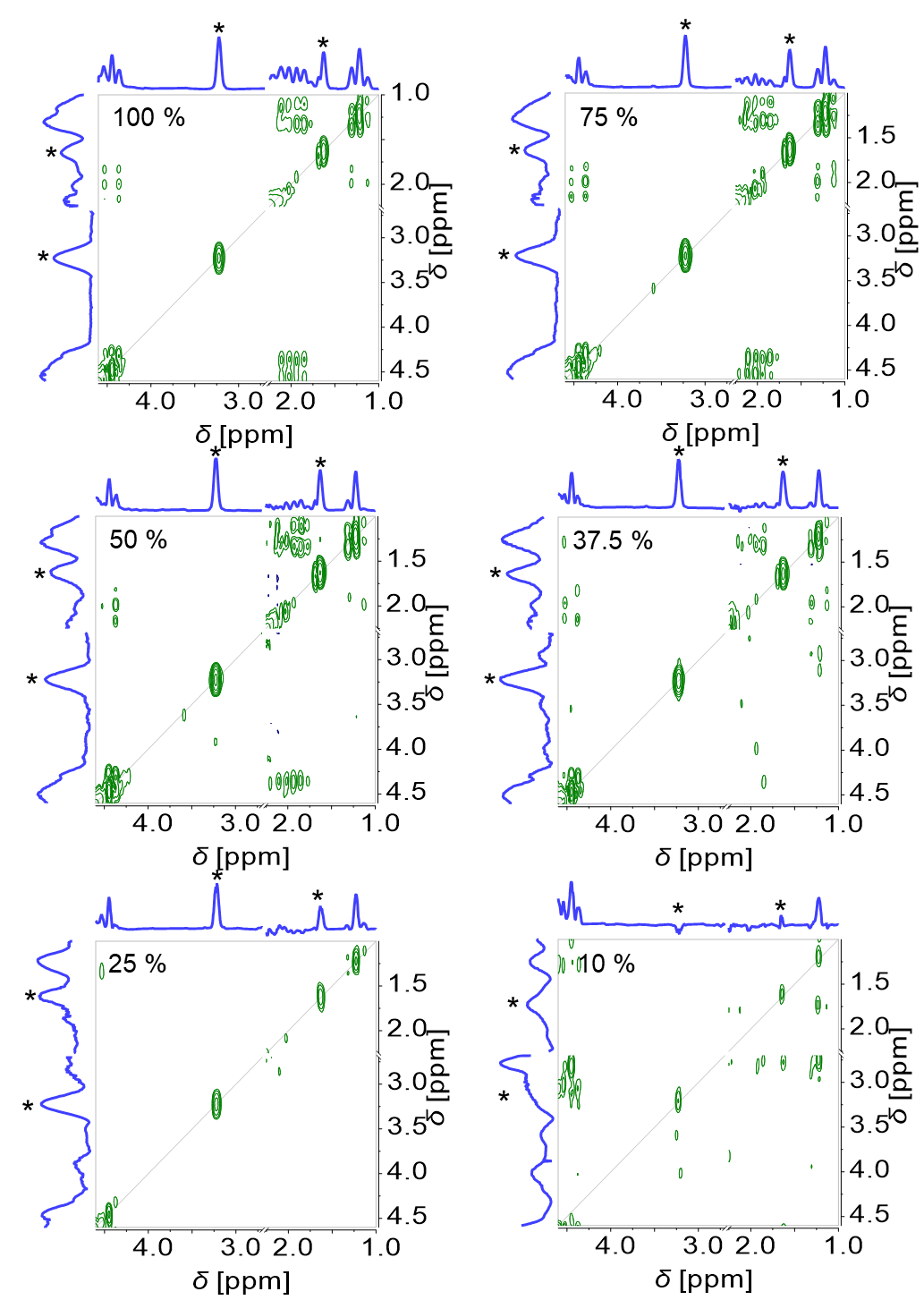


**Figure S10.** Aliphatic region of the COSY spectrum of propyl paraben (30 g L^-1^ in acetone/water 60/40 v/v) at 0.4 mL min^-1^ with circulatory setup with 128 increments in the indirect dimension, recorded with different NUS densities (with acetone signal cut out for display through an axis break at 2.25-2.70 ppm). * indicates an acetone signal ^13^C satellite.


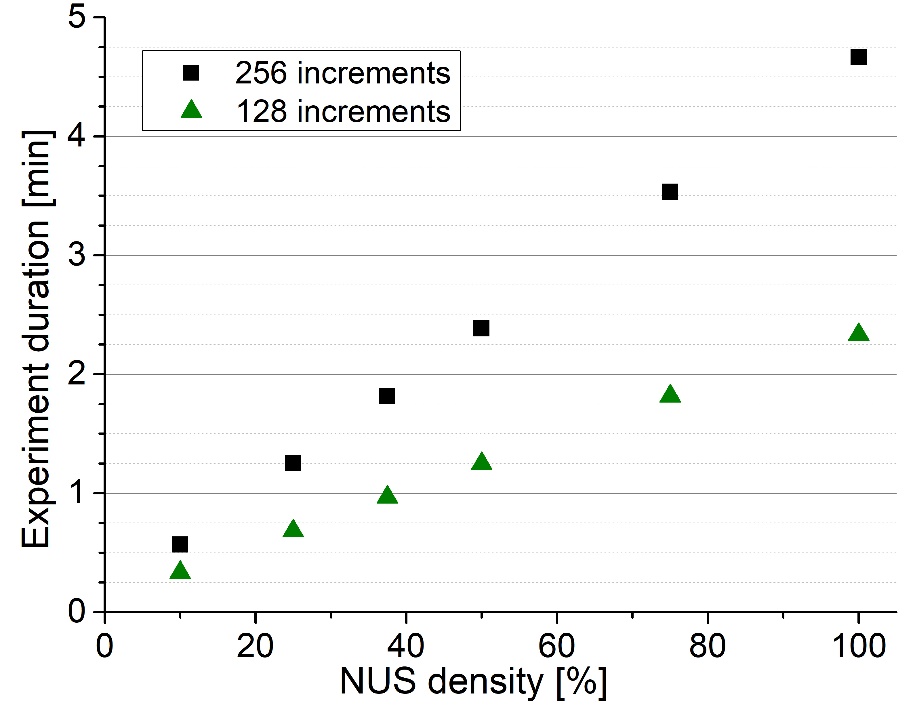


**Figure S11.** Experiment durations for COSY-NUS experiments at Ernst condition and 0.4 mL min^-1^. Experiment durations for 37.5 % NUS density are 1 min 2 s with 128 increments in the indirect dimension and 1 min 54 s with 256 increments in the indirect dimension.

# S.5. Online coupling of HPLC and 2D-NMR with stop-flow

### S5.1. Feasibility with propyl paraben – with 128 increments in indirect dimension


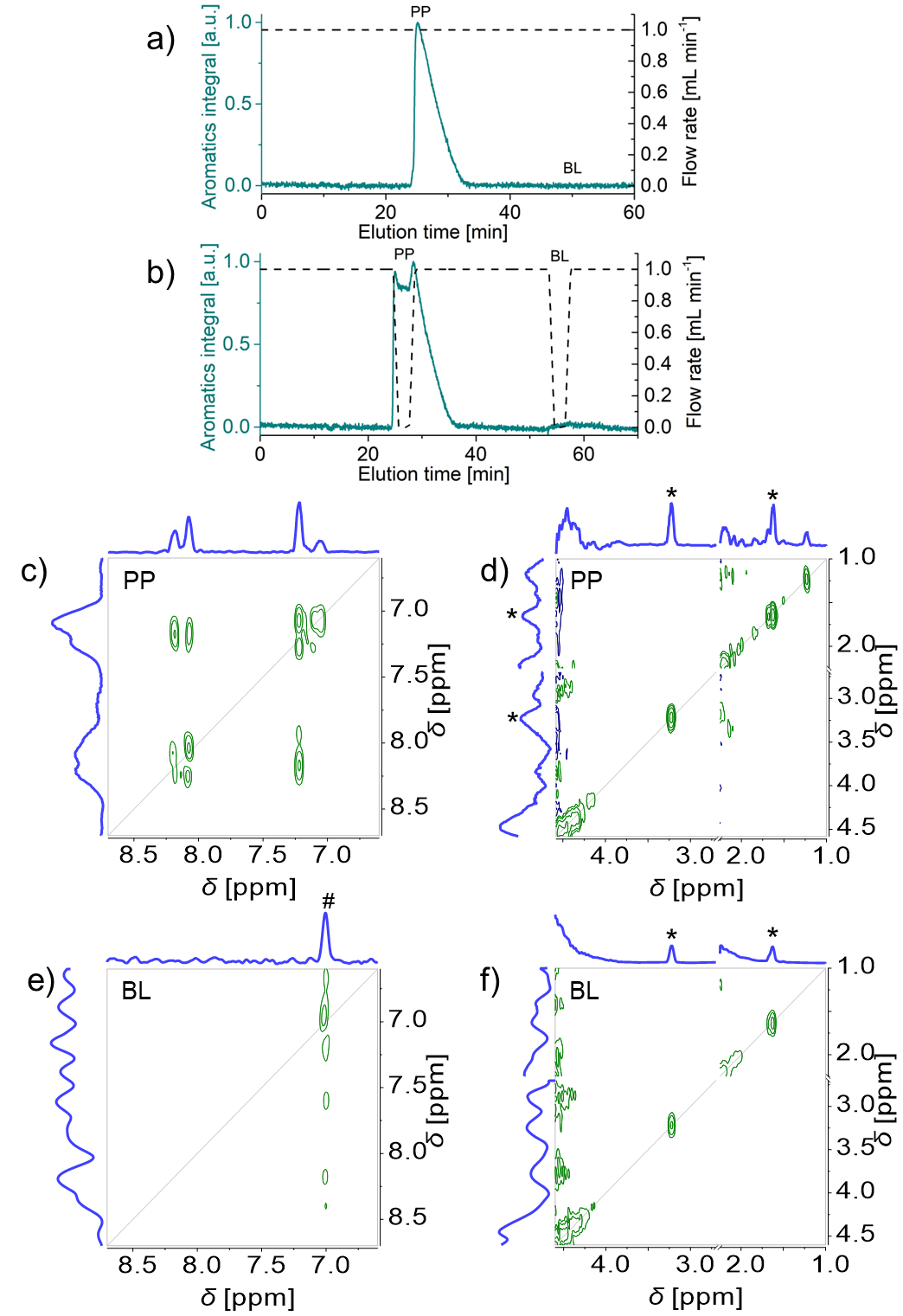


**Figure S12.** HPLC-benchtop NMR analysis with stop-flow for propyl paraben PP (30 g L^-1^ in acetone/water 60/40 v/v): ^1^H NMR elugrams and corresponding flow rate profiles (a, b), COSY spectra (c-f). ^1^H NMR elugrams and corresponding flow rate profiles at constant flow rate (a) were used to determine where to add the stop-flow increments in indirect dimension (b) through a 1 min ramp down, a 2 min stop, and a 1 min ramp back up. COSY spectra (at Ernst condition, with 1 scan, 128 increments in indirect dimension, 37.5 % NUS density, in 1 min) recorded during the stop in PP peak (c, d) and in baseline (BL) (e, f). Spectra are shown covering the aromatic region (c, e) and the aliphatic region (d, f, with axis break at 2.25-2.70 ppm). * indicates an acetone signal ^13^C satellite and ^#^ an intermodulation artifact.

### S5.2. Separation of parabens

**
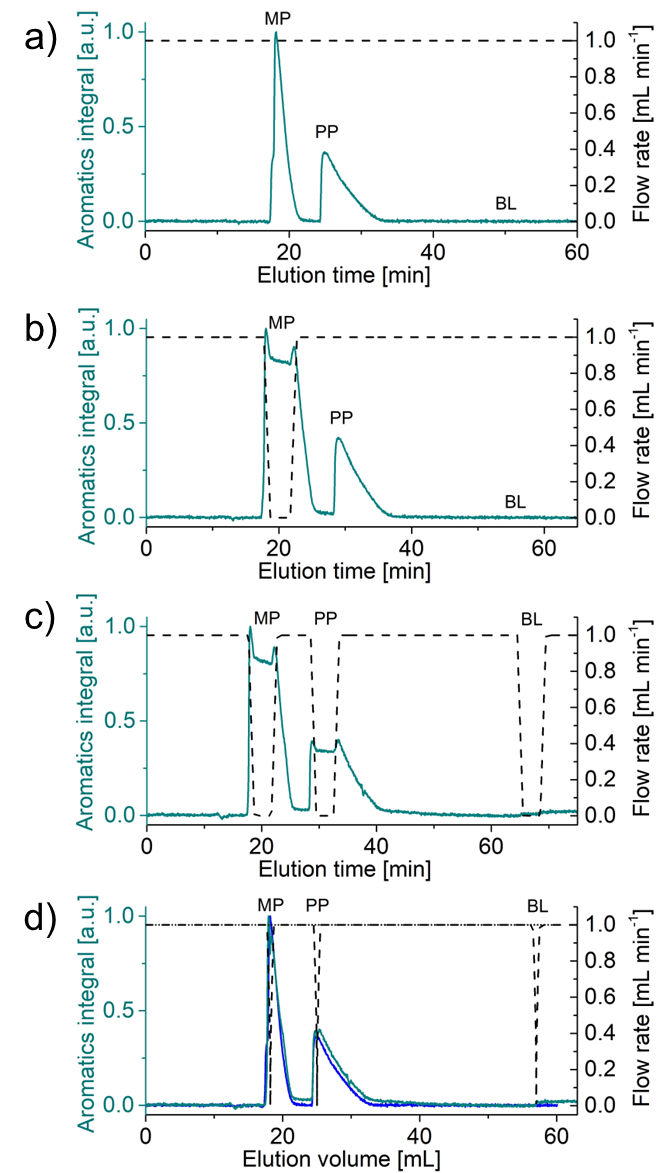
**

**Figure S13.** ^1^H NMR elugrams and corresponding flow rate profiles recorded in preparation for stop-flow experiments for the separation and characterization of a mixture of methyl paraben MP and propyl paraben PP (30 g L^-1^ each in acetone/water 60/40 v/v), with a constant flow rate of 1 mL min^-1^ except for the stops (1 min ramp down, 3 min stop, 1 min ramp back up): no stop (a), stop at maximum of MP peak (b), stops at maxima of MP and PP peaks and in baseline (BL) (c). ^1^H NMR elugrams and corresponding flow rate profiles obtained without stop (a) and with 3 stops (c) are overlayed as a function of elution volume in (d).

### S5.3. COSY spectra recorded in the baseline


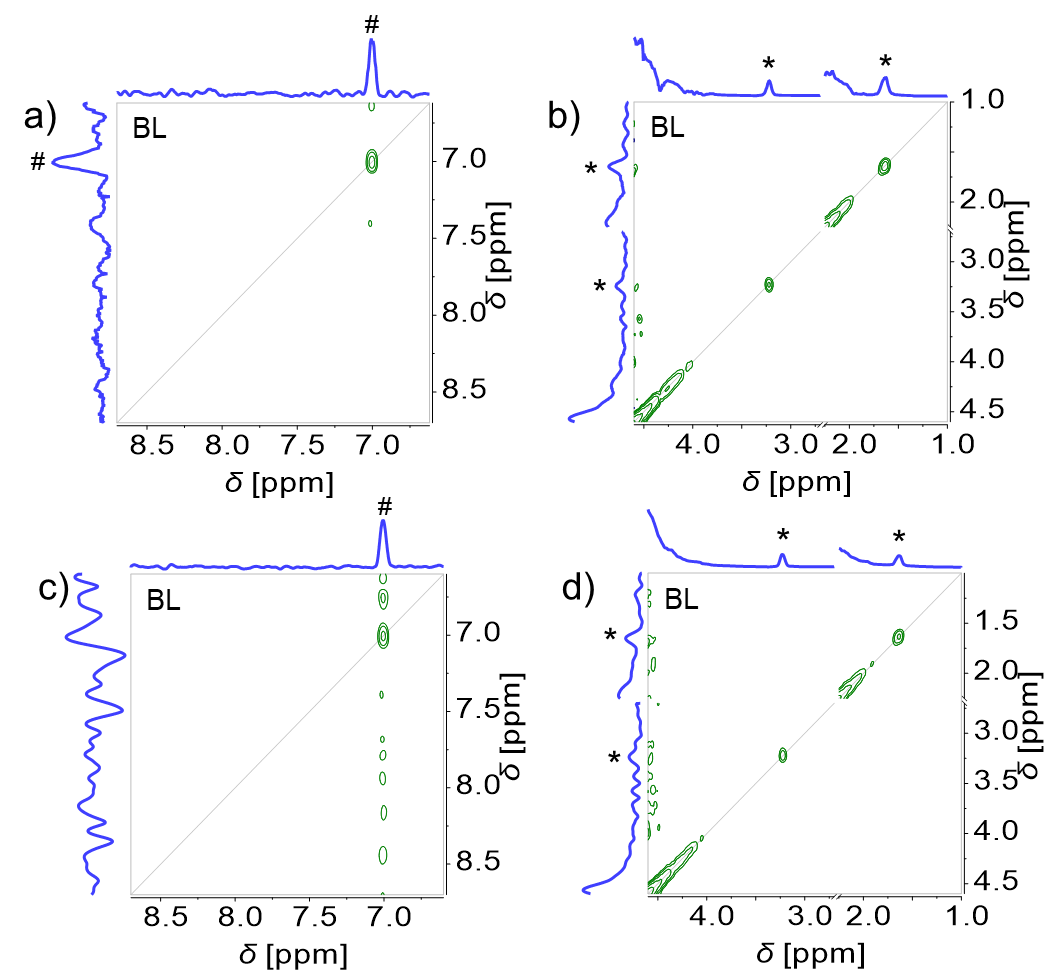


**Figure S14.** COSY spectra recorded during a stop in the baseline BL during HPLC-benchtop 2D-NMR analysis with stop-flow for propyl paraben PP (a,b) and for a mixture of methyl paraben MP and PP (c,d), at 50 g L^-1^ each in acetone/water 60/40 v/v. See Figure 3 and 4 for the corresponding flow profiles and COSY spectra parameters. COSY spectra are shown covering the aromatic region (a, c), and the aliphatic region (b, d, with acetone signal cut out for display through an axis break at 2.25-2.70 ppm). * indicates an acetone signal ^13^C satellite and # an intermodulation artifact.

# S6. Online coupling of HPLC and 2D-NMR with slow-flow


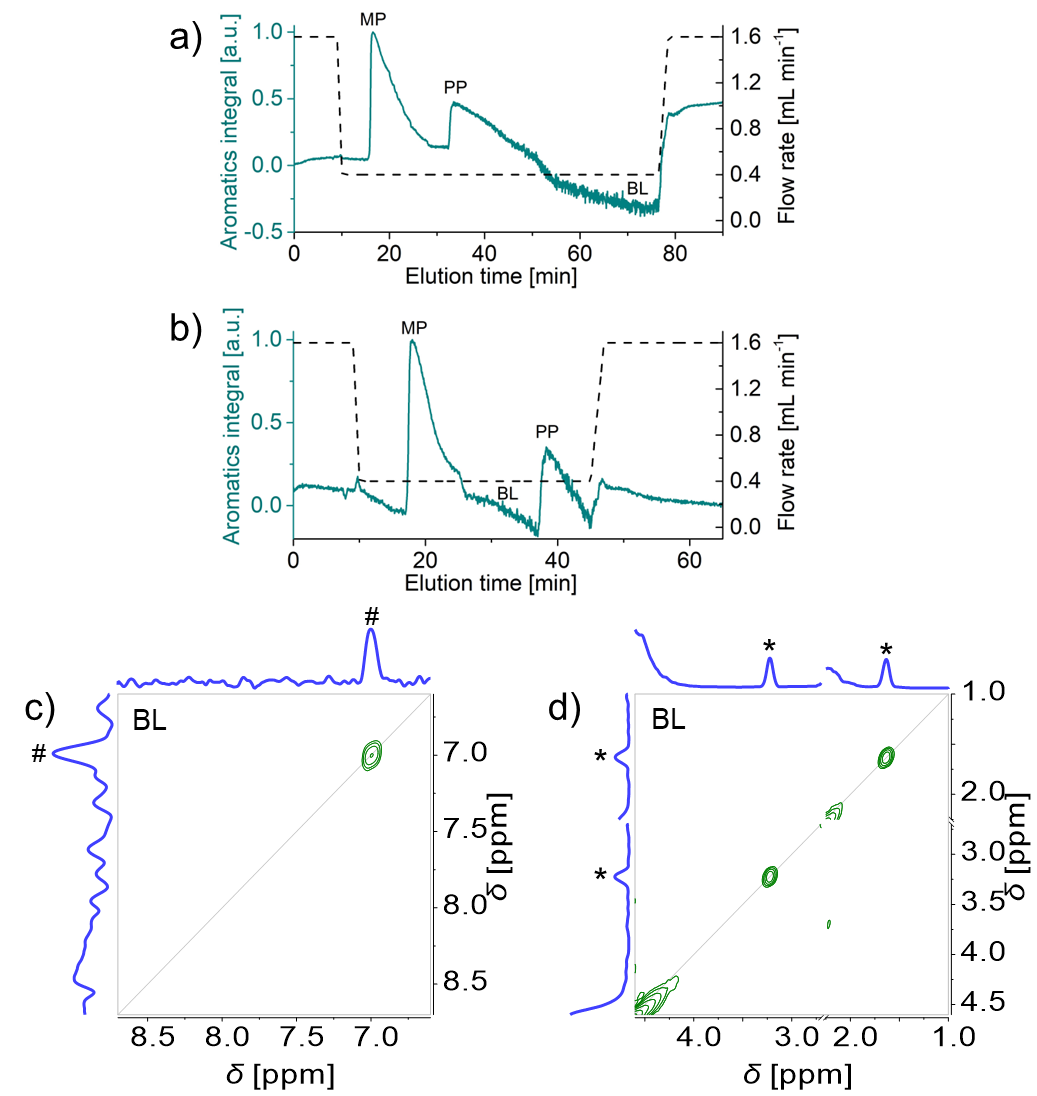


**Figure S15.** HPLC-benchtop 2D-NMR analysis in slow-flow mode for a mixture of methyl paraben MP and propyl paraben PP (50 g L^-1^ each in acetone/water 60/40 v/v): ^1^H NMR elugrams and corresponding flow rate profiles (a, b), COSY spectra recorded in the baseline BL (c, d). ^1^H NMR elugrams and corresponding flow rate profiles , show a flow rate of 1.6 mL min^-1^, slowed down to 0.4 mL min^-1^ (with 1 min ramp down and 2 min ramp up) over the peaks elution volume ranges: 15 to 42 mL (a) or 15 to 30 mL (b). See Figure 5 for the COSY spectrum parameters shown here covering the aromatic region (c), and the aliphatic region (d), with acetone signal cut out for display through an axis break at 2.25-2.70 ppm). * indicates an acetone signal ^13^C satellite and # an intermodulation artifact.

A slight shift in elution times, baseline drifts and noise are attributed to a drift of the magnetic field after the flow rate change from 1.6 to 0.4 mL min^-1^. This will be discussed in a further publication currently in preparation by our team, including insulation strategies to minimize it.

# S7. Online coupling of HPLC and 2D-NMR at steady-flow


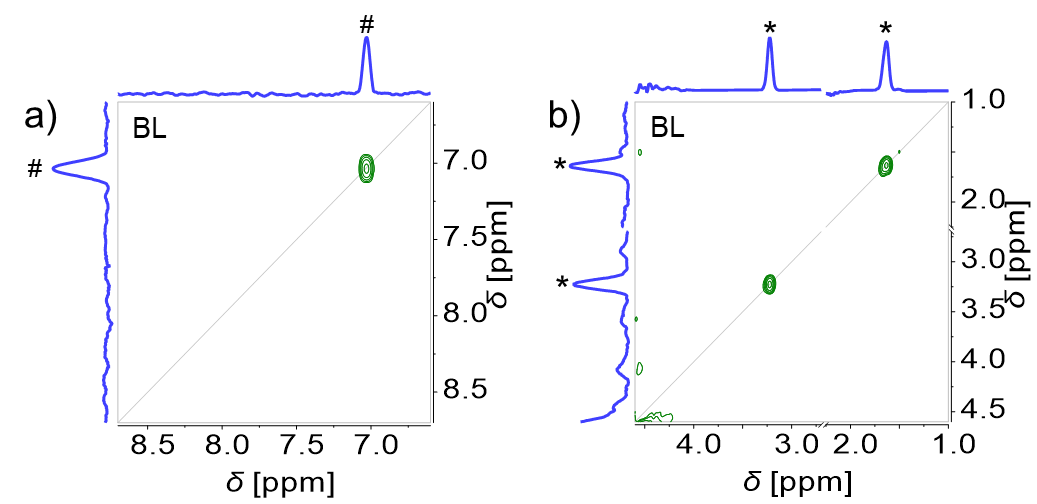


**Figure S16.** COSY spectrum recorded in the baseline BL during HPLC-benchtop 2D-NMR analysis in steady-flow mode at 1 mL min^-1^ for a mixture of methyl paraben MP and propyl paraben PP (50 g L^-1^ each in acetone/water 60/40 v/v). See Figure 6 for the COSY spectrum parameters. COSY spectrum is shown covering the aromatic region (a), and the aliphatic region (b), with acetone signal cut out for display through an axis break at 2.25-2.70 ppm). * indicates an acetone signal ^13^C satellite and # an intermodulation artifact.

# S8. NMR sensitivity of the HPLC-benchtop 2D-NMR coupling

**Table S1.** Signal-to-noise ratio (*SNR*) values for the cross-peak at 8.05,7.20 ppm in the COSY spectra of methyl paraben (MP) and propyl paraben (PP) recorded with HPLC-benchtop 2D-NMR in stop-flow, slow-flow and steady-flow modes. See Figures 3 to 6 and experimental section for experimental conditions.

| Coupling mode | Injected sample | *SNR* for MP peak | *SNR* for PP peak |
| --- | --- | --- | --- |
| Stop-flow | MP + PP mixture | 394, 349 | 164, 173 |
| Slow-flow | MP + PP mixture | 352, 370 | 81, 121 |
| Steady-flow | MP + PP mixture | 244, 256 | 77, 85 |
| Stop-flow | PP | - | 139, 143 |

# Reference

[1] K. Albert, "Chapter 1 - LC-NMR: theory and experiment", in *On-line LC-NMR and Related Techniques*, John Wiley & Sons Ltd., Chichester, UK, **2002**, p. 1.
